# Epidermal growth factor receptor signaling protects epithelia from morphogenetic instability and tissue damage in *Drosophila*

**DOI:** 10.1242/dev.201231

**Published:** 2023-03-09

**Authors:** Kentaro Yoshida, Shigeo Hayashi

**Affiliations:** ^1^ Laboratory for Morphogenetic Signaling, RIKEN Center for Biosystems Dynamics Research, 2-2-3 Minatojima-minamimachi, Chuo-ku, Kobe, Hyogo 650-0047, Japan; ^2^ Department of Biology, Kobe University Graduate School of Science, 1-1 Rokkodai-cho, Nada-ku, Kobe, Hyogo 657-8051, Japan

**Keywords:** Morphogenesis, Invagination, Apoptosis, Wound healing, Vitelline membrane, Epidermal growth factor signaling

## Abstract

Dying cells in the epithelia communicate with neighboring cells to initiate coordinated cell removal to maintain epithelial integrity. Naturally occurring apoptotic cells are mostly extruded basally and engulfed by macrophages. Here, we have investigated the role of Epidermal growth factor (EGF) receptor (EGFR) signaling in the maintenance of epithelial homeostasis. In *Drosophila* embryos, epithelial tissues undergoing groove formation preferentially enhanced extracellular signal-regulated kinase (ERK) signaling. In EGFR mutant embryos at stage 11, sporadic apical cell extrusion in the head initiates a cascade of apical extrusions of apoptotic and non-apoptotic cells that sweeps the entire ventral body wall. Here, we show that this process is apoptosis dependent, and clustered apoptosis, groove formation, and wounding sensitize EGFR mutant epithelia to initiate massive tissue disintegration. We further show that tissue detachment from the vitelline membrane, which frequently occurs during morphogenetic processes, is a key trigger for the EGFR mutant phenotype. These findings indicate that, in addition to cell survival, EGFR plays a role in maintaining epithelial integrity, which is essential for protecting tissues from transient instability caused by morphogenetic movement and damage.

## Teaser

**Highlighted Article:** EGF receptor signaling in *Drosophila* protects epithelial tissues under mechanical stress from collapsing by preventing apical cell extrusion.

## INTRODUCTION

The maintenance of epithelial integrity is essential for animal development and homeostasis. Cell proliferation often produces excess cells that are removed at a later point from the epithelium to maintain optimal cell numbers. The morphogenetic movement of epithelial folding or cell overcrowding applies mechanical stress to the cells to trigger programmed cell death (Eisenhoffer et al., 2012; Levayer et al., 2016; Marinari et al., 2012). These cells are extruded by the contractile activity of healthy neighboring cells. Stabilization of stressed epithelia and rapid repair of epithelial damage due to cell death are especially important in the rapid embryogenesis of *Drosophila* (22 h) compared with zebrafish (72 h) or mouse (3 weeks).

*Drosophila* embryogenesis starts from the syncytium, where diploid nuclei fill the surface area and the yolk occupies the interior. *Drosophila* embryos are internally pressurized, and their surfaces are supported by the vitelline membrane. Poking a hole in the vitelline membrane causes rapid cytoplasmic leakage; embryos do not survive after vitelline membrane removal. Embryonic morphogenesis proceeds via epithelial folding and invagination in the basal (internal) direction against internal pressure, by increasing the apical surface tension of the epithelium, which drives inward tissue movement (Lecuit and Lenne, 2007). Although both apical and basal extrusions have been documented for dying cells in tissue culture and vertebrate tissues (Katoh and Fujita, 2012), *Drosophila* embryos almost always extrude dying cells basally. Here, phagocytic macrophages engulf apoptotic cells so that toxic cell debris is quickly removed and digested for recycling cellular components (Abrams et al., 1993). How the delamination of dying cells is directed towards the interior of the embryo against internal pressure is not understood.

Programmed cell death is induced by various cellular stresses and cell mis-specification in mutants of pattern formation genes (Abrams et al., 1993). One major pro-survival system is epidermal growth factor receptor (EGFR) signaling, which regulates extracellular signal-regulated kinases (ERK) signaling in highly dynamic spatiotemporal patterns (Gabay et al., 1997). Massive cell death was observed in EGFR mutants (Price et al., 1989; Schejter and Shilo, 1989). Although EGFR is ubiquitously expressed (Revaitis et al., 2020), the onset of extensive cell death in EGFR mutants is limited to the epidermis of the head and ventral thorax, suggesting that additional factors contribute to the specificity of the prosurvival function of EGFR (Clifford and Schüpbach, 1992). In addition, EGFR supports the survival of potentially apoptotic cells in mutants of segmentation genes (Crossman et al., 2018) and promotes the repair of the epithelial opening after apoptotic cell extrusion (Valon et al., 2021).

In this study, we address the role of EGFR signaling in the maintenance of epithelial integrity in embryos. We show that EGFR is preferentially activated in the grooves of epithelia, where the apical cell surface is detached from the vitelline membrane. We show that EGFR and the vitelline membrane, which provides apical mechanical support, coordinately protect apoptotic epithelial cells from extrusion into the apical side. Here, we discuss the mechanism that prevents embryonic epithelial tissues from collapsing under the mechanical stress produced by rapid morphogenetic movements.

## RESULTS

### EGFR-dependent ERK activity is elevated in the region of epithelial morphogenesis

To reveal the dynamic activation patterns of EGFR during *Drosophila* embryogenesis, we monitored the Förster resonance energy transfer (FRET) probe of ERK activity (Aoki et al., 2017; Komatsu et al., 2011; Ogura et al., 2018). The FRET activity showed spatiotemporal patterns in the epidermis (Fig. S1A,C,E,G). Mutation of *rhomboid* (*rho*), which encodes the key protease required for the activation of the major EGFR ligand Spitz (Shilo, 2016), greatly reduced FRET activity (Fig. S1B,D,F,H). These results suggest that the FRET activity reflects the activation of EGFR-ERK signaling. The FRET activity was also observed in mitotic cells due to its sensitivity to Cdk1 (Aoki et al., 2013), independently of *rhomboid* (Fig. S1D, asterisks).

**Fig. 1.**
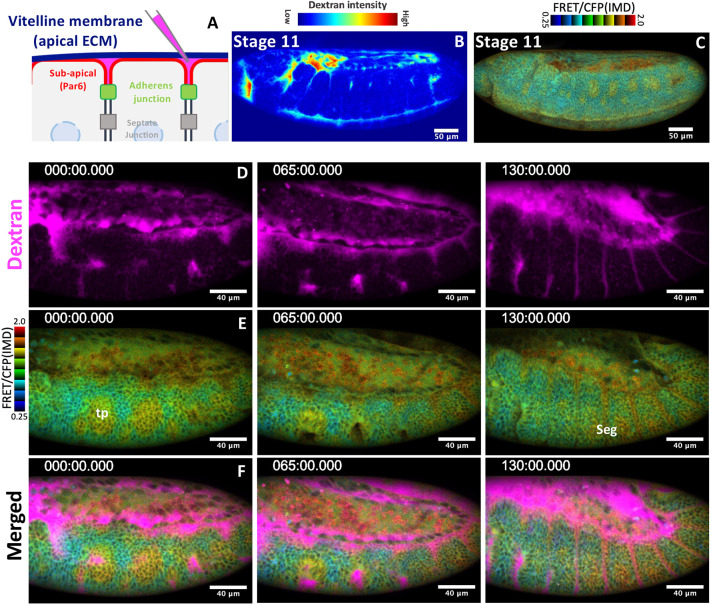
**EGFR-dependent ERK activity is elevated in the region of epithelial morphogenesis.** (A) Procedure of perivitelline space labeling. (B,C) The imaging of the perivitelline space (B) and ERK-FRET activity (C) in a stage 11 embryo (*n*=5). (D-F) Simultaneous live-imaging of the perivitelline space and ERK-FERT activity (Movie 1). Time (t)=0 was set at the time of tracheal invagination of T1 segment. Times are in minutes. tp, tracheal pit; seg, segmental groove.

To further clarify the relationship between EGFR signaling and morphogenetic movement, fluorescent dextran was injected in the space between the vitelline membrane and epidermal cells (perivitelline space, Fig. 1A). A high dextran signal was detected in the head-thorax boundary stomodeum (mouse cavity), and ventral midline and tracheal pit, where active epithelial folding forms a space between the epithelia and the vitelline membrane (Fig. 1B). Live imaging showed that ERK activity was upregulated during epithelial morphogenesis of the tracheal pit and segmental grooves (Fig. 1C-F, Movie 1). In addition, perivitelline gaps were detected in the place above mitotic cells (Fig. S1I,I′). Although the high FRET ratio in the amnioserosa was not reduced in *rhomboid* mutants (Fig. 1F,H), the disintegration of amnioserosa was observed in EFGR mutants (Nishimura et al., 2007; Shen et al., 2013). These results indicate that EGFR signaling is activated in epithelial tissues undergoing morphogenesis.

### Apical cell extrusion and epithelial collapse in EGFR mutant embryos

The terminal phenotype of zygotic EGFR mutants includes massive cell death and loss of a large part of the ventral cuticles, suggesting that the bulk of epidermal cells failed to secrete the cuticle or were lost (Clifford and Schüpbach, 1989; Price et al., 1989; Schejter and Shilo, 1989). Observation of all stages up to stage 11 revealed that the bulk shape of the embryo was maintained; however, various local phenotypes existed, such as defects in ventral epidermal patterning and tracheal invagination (Nishimura et al., 2007; Raz and Shilo, 1993).

**Fig. 2.**
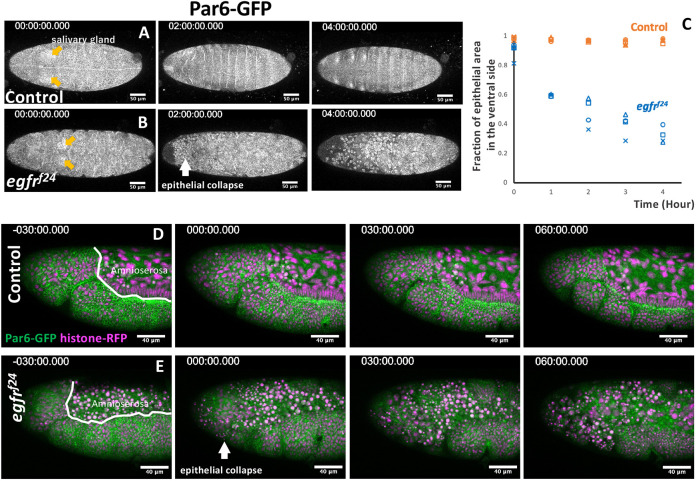
**Epithelial collapse in the EGFR mutant embryos.** (A,B) Time-lapse imaging of ventral views of control (A) and EGFR-mutant (B) embryos carrying the junction marker Par6-GFP. Time (t)=0 was set at the time of salivary gland invagination (orange arrows). Times are in minutes. The site of the initial epithelial collapse in the EGFR mutant is marked with a white arrow (t=2 h). (C) Time course of the loss of junctional Par6-GFP localization. *n*=4 for each genotype. (D) Lateral view of the head of a control embryo. (E) An EGFR mutant embryo. Amnioserosa had already disintegrated at t=−30 min, followed by the loss of epithelial integrity at t=0 min. At t=60 min, the entire head region collapsed (*n*=5).

To understand the process of the massive loss of epidermal cells in EGFR mutants, we performed time-lapse imaging of EGFR mutant embryos. Ventral views of the control embryos showed segment groove formation (t=2 h) and head involution (t=4 h) (Fig. 2A). EGFR mutants at early stage 11 (t=0) showed normal epithelial integrity, as shown by the junctional localization of Par6-GFP and the clustering of salivary gland placode cells (Fig. 2B, orange arrows). At the germ band retraction stage, several condensed cells lacking junctional Par6-GFP appeared in the head region (Fig. 2B; t=2 h, white arrow), and this phenotype expanded posteriorly. After 4 h, the bulk of the ventral epidermis (∼70%) lost junctional Par6-GFP (Fig. 2B, t=4 h, Fig. 2C).

Time-lapse imaging performed in a lateral view (Movie 2) showed that the amnioserosa was the first to lose continuity in EGFR mutants (Fig. 2D,E, t=−30 min, Movie 2) (Shen et al., 2013). After the onset of tracheal invagination at stage 11, rounded cells were frequently observed on the apical side of the epidermis (Fig. 2E, t=0, 30 min). Subsequently, the entire head ectoderm of the EGFR mutants lost epithelial connections and collapsed (Fig. 2E, t=60 min, arrows), whereas the thoracic ectoderm retained its epithelial state.

### Apical cell extrusion from apoptotic cell clusters in EGFR mutants

**Fig. 3.**
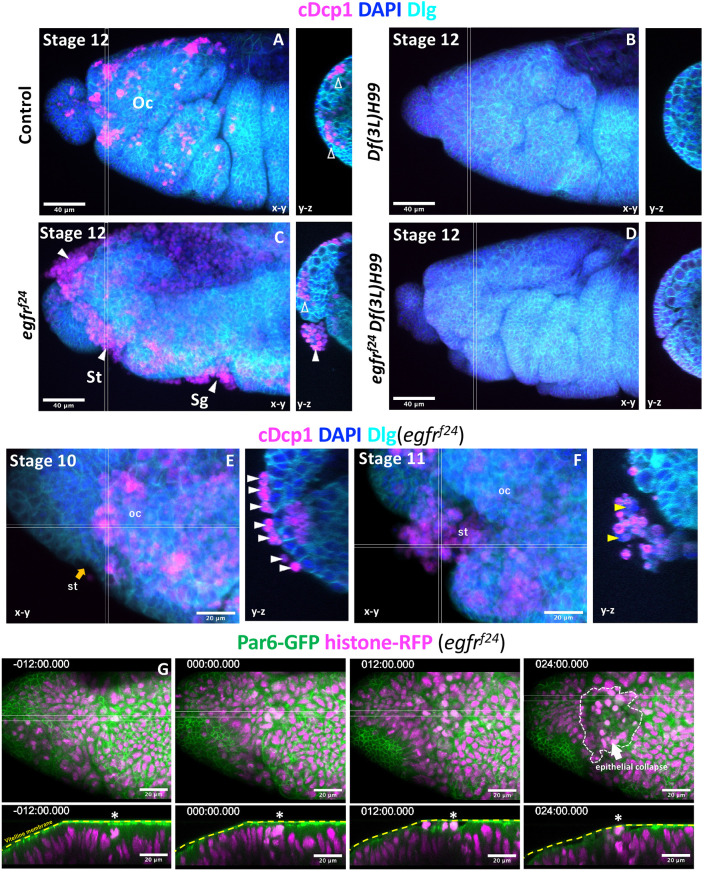
**Apical extrusion of apoptotic and non-apoptotic cells in EGFR mutants.** (A-F) Active caspase staining (cDcp1) of a control (A), a Df(3L)H99 mutant (B), an EGFR mutant (C,E,F) and an EGFR/Df(3L)H99 double mutant (D). More than 10 embryos of each genotype were examined, except for the EGFR/Df(3L)H99 double mutant (*n*=4). cDcp1-positive cells appeared as clusters within, or basal to, the plane of the epithelium (open arrowheads) of control embryos (A). In EGFR mutants, apoptotic cells appeared basal to the epithelium; however, most appeared apical to the epithelium (white arrowheads) (C). Apical cells were all cDcp1 positive at early stage 10 (E). At late stage 11, cDcp1-negative cells were found in the apical location (yellow arrowheads, F). (G) Live imaging of the head region of the EGFR mutant showing an extensive collapse in the ocular segment of the head (arrow in the 24 min panel). Here, t=0 was set at the time the first apical cell extrusion event was observed (asterisks). st, stomodeum; oc, ocular segment; sg, salivary gland. Orange arrow indicates the invagination region of ectoderms into the stomodeum.

We next asked what condition makes the head especially sensitive to the EGFR mutation, by looking at the relationship between the epithelial collapse phenotype and the pattern of apoptosis and morphogenesis. Apoptosis in the head is developmentally controlled by the head patterning Hox gene *deformed* (*dfd*), which stimulates the expression of the proapoptotic gene *reaper* (Lohmann et al., 2002; Nassif et al., 1998). At stage 12, a number of apoptotic cells, which were marked by cleaved Drosophila caspase 1 (cDcp1), were present the ocular segment, distributed as clusters in the plane of the ectoderm or on the basal side (Fig. 3A, open arrowheads). The number of apoptotic cells was drastically reduced in the *Df(3L)H99* mutant, in which four major proapoptotic genes are removed (Fig. 3B). In EGFR mutant embryos, a large number of apoptotic cells occupied the apical area of grooves corresponding to the ocular segment, stomodeum and salivary gland (Fig. 3C, closed arrowheads). *Egfr* and *Df(3L)H99* double-mutant embryos showed very few apoptotic cells and no tissue disintegration (Fig. 3D). These results indicate that apoptosis is required for tissue disintegration in EGFR mutant embryos.

Observation of the ocular segment of EGFR mutants in early stage 10 showed that apically extruded cells were individually attached to the surface of the epithelium, and these cells were all cDcp1 positive (Fig. 3E). In stage 11 embryos after stomodeum invagination, clusters of extruded cells, including both cDcp1-positive and -negative cells, were observed (Fig. 3F, yellow arrowheads).

Live imaging of the EGFR mutant heads was performed (Fig. 3G, Movie 3). Before apical cell extrusion, the apical cell surface was tightly juxtaposed to the vitelline membrane (Fig. 3G, t=−12 min). A gap between the apical cell surface and vitelline membrane appeared (Fig. 3G, t=0). One cell with condensed chromatin was found to move into the apical space (Fig. 3G, asterisk), followed by the extrusion of additional cells (white arrowhead), leading to the collapse of almost the entire head (Fig. 3G, t=24 min).

Mitotic cell staining showed that mitosis occurred in clusters within the planes of epithelia in the heads of both control and EGFR mutant embryos (Fig. S2A,B). We also observed no mitotic cells in the cluster of apically extruded cells of the EGFR mutants (Fig. S2B).

### Apical cell extrusion at the site of epithelial detachment from the vitelline membrane

**Fig. 4.**
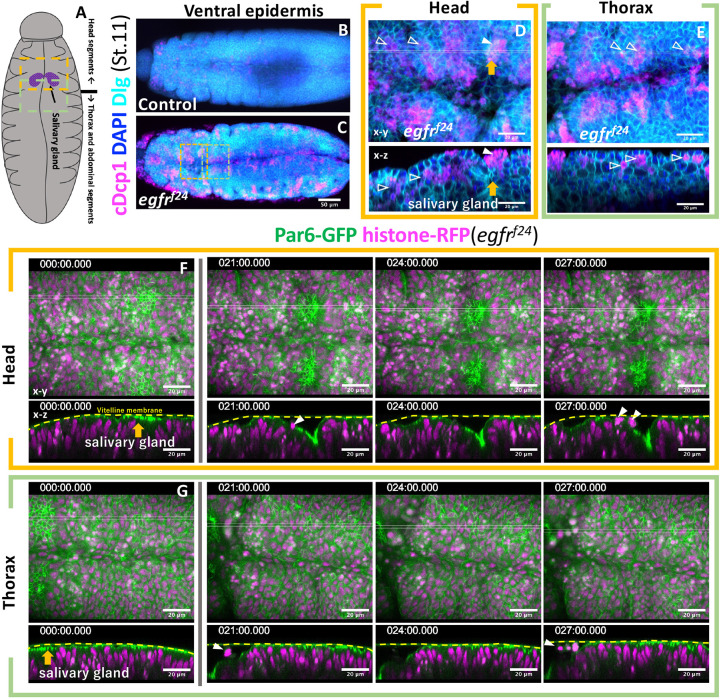
**Apical cell extrusion at the site of vitelline membrane detachment.** (A) Schematic of the ventral view of stage 11 embryos. (B-E) Active caspase staining (cDcp1) of control (B) and EGFR mutant (C) embryos. Parts of the head and thoracic regions of C (dotted box) are enlarged in D and E. Unfilled arrowheads, basally extruded cells; filled arrowheads, apically extruded cells; orange arrows, salivary gland invagination. (F,G) Time-lapse series of the ventral head and thorax imaged from two separate embryos, showing apical cell extrusion in the invaginating salivary gland primordia (orange arrows and white arrowheads). Other parts maintained tight vitelline membrane contact at this stage. Times are in minutes.

To further clarify the relationship between epithelial fold formation and apical cell extrusion, we further investigated posterior segments (Fig. 4A). A ventral view of stage 11 embryos, before the onset of the complete collapse of the ventral epidermis, showed a significantly increased number of apoptotic cells forming clusters in EGFR mutants (Fig. 4B,C). Cross-sectional views of mutant embryos showed that most apoptotic cell clusters were found on the basal side of the epithelia (Fig. 4D,E, open arrowheads). A notable exception was the salivary gland placode, where apoptotic cells formed clusters on the apical side (Fig. 4D,E, orange arrow, closed arrowhead). Time-lapse imaging showed that apical cell extrusion occurred in the salivary gland pit after the invaginating placode formed an open space between the vitelline membrane (Fig. 4F,G, Movie 4). No apical cell extrusion was observed outside the salivary gland placode in the imaged region of mutant embryos at this stage.

In order to detect any change in cellular adhesion, we monitored the expression of the adherens junction marker E-cadherin in the salivary gland placode (Fig. S2C,D). In control, those cells adopted an elongated shape and directed long cell boundaries toward the site of invagination. In EGFR mutants, epithelial cells adopted a more irregular shape. Notably, frequent gaps of E-cadherin staining in the cell boundary were observed (Fig. S2D, yellow dots). No prominent gaps in the subapical region (Par6-GFP, Figs 3G and 4F,G) or in the lateral contact site (Dlg, Fig. 4D-G) were observed. These results suggest that the gap phenotype is specific to the adherens junction, which is anchored to the cytoskeletons.

### Forced detachment of the vitelline membrane caused the rapid collapse of EGFR mutant epithelia

Considering the results thus far, we reasoned that attachment to the vitelline membrane suppresses epithelial instability under the loss of EGFR function. To test this hypothesis, we observed epithelia mechanically detached from the vitelline membrane.

**Fig. 5.**
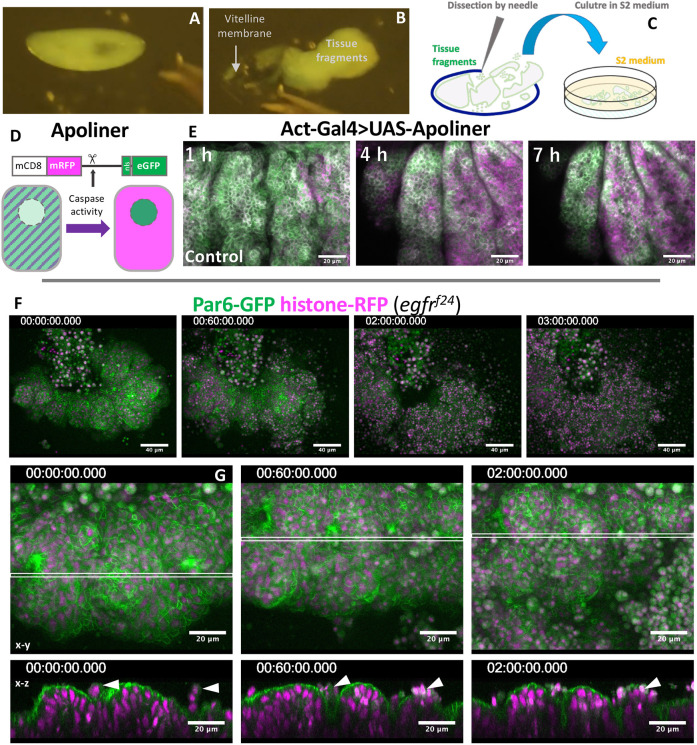
**Rapid collapse of EGFR tissue fragments in explant cultures.** (A-C) The procedure of embryo dissection. (D) Apoliner caspase activity detector. (E-G) Time-lapse imaging of cultured tissue fragments. (E) Apoliner activity in a control tissue fragment. (F) An EGFR mutant tissue fragment undergoing rapid disintegration. (G) The early phase of EGFR mutant tissue culture, showing frequent apical cell extrusion (arrowheads). Imaging started within 1 h of dissection (time is h:min:s).

A stainless steel needle was used to remove the vitelline membrane from stage 11 embryos, and the resultant embryo fragments were cultured in a petri dish (Fig. 5A-C). EGFR^+^ tissue fragments expressing the caspase activity reporter apoliner (Fig. 5D, Bardet et al., 2008) showed a gradual increase in the signal at ∼4 h, but their tissue structure mostly remained intact over 7 h (*n*=13 cultured fragments, Fig. 5E). The tracheal placode maintained the ERK-FRET activity at least 60 min after devitellinization, and the FRET activity declined thereafter (Movie 5). Strikingly, when EGFR mutant epithelial tissues were cultured, they rapidly lost integrity and almost completely collapsed within 5 h (*n*=13 cultured fragments, Fig. 5F, Movie 6). Cross-sectional views showed that EGFR mutant epidermal fragments were folded and extruded several cells apically before completely collapsing (Fig. 5G).

### EGFR signaling is required for protecting the epidermis from tissue wound

**Fig. 6.**
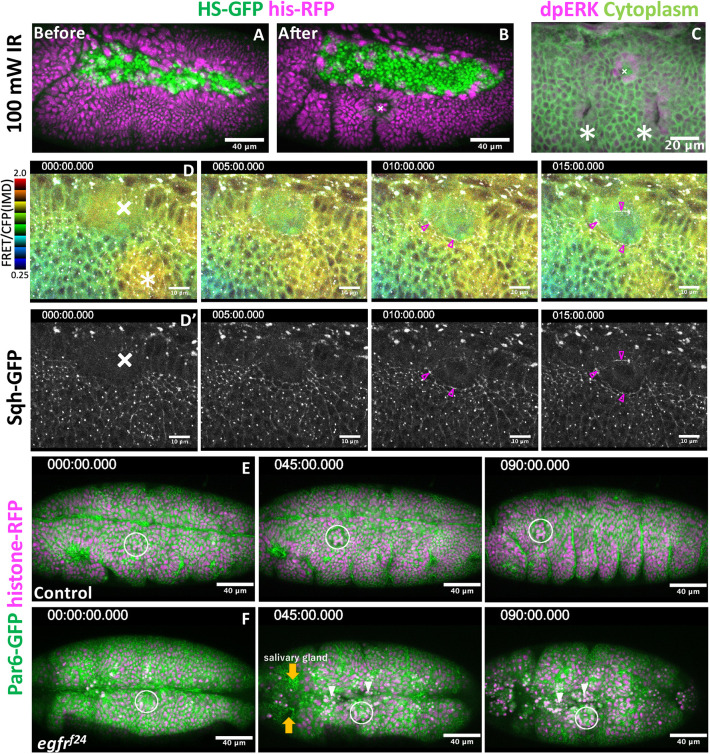
**EGFR signaling is required for tissue repair.** (A,B) Tissue wounds treated with infrared laser illumination. (C) ERK activation in the vicinity of the wound region. Crosses, wound site; asterisks, tracheal placode. (D,D′) Simultaneous recording of ERK activity (FRET probe, D) and myosin activity (D′). Magenta arrowheads indicate myosin accumulation around wound region. (E,F) Laser wound damage is limited locally in the control group (E). Tissue disintegration spreads from the wound site in EGFR mutants (F). White circles indicate the wound site. Orange arrows indicate invagination of the salivary gland. White arrowheads indicate apical cell extrusion around wound region. Times are in minutes.

Next, we investigated whether EGFR protects tissues from injury. To achieve this, we used an approach to induce epithelial lesions with an infrared (IR) laser that reaches deep into the target and avoids the risk of damaging the vitelline membrane (Kato et al., 2016). Laser irradiation caused a wound (Fig. 6A,B; Miao and Hayashi, 2015) and induced the expression of di-phosphorylated ERK in one or two cell rows around the wounded region, as previously observed in tissue culture cells and *Drosophila* (Fig. 6C, Matsubayashi et al., 2004, Hiratsuka et al., 2015; Geiger et al., 2011; Li et al., 2013; Mace et al., 2005; Wang et al., 2009). Under the same conditions, the ERK-FRET reporter was immediately activated in a region covering the adjacent segment and tracheal placode 5 min after wounding (Fig. 6D, 5 min). In the next 10 min, broad ERK activity was reduced to one or two cell rows at the wound edge, and this activity was sustained for at least 30 min after wounding (Fig. 6D, 10-15 min, Movie 7). Cables of myosin appeared at the wound edge 10 min after wounding (Fig. 6D′).

Having established the conditions for laser wounding, we next applied this technique to EGFR mutants. A previous wounding experiment of stage 15 embryos demonstrated that wound healing was delayed in EGFR hypomorphic mutants (Geiger et al., 2011). Here, we chose to create a wound in the region requiring EGFR (ventral epidermis), i.e. at the left side of first thoracic segment in stage 11, where we would not see any defect in EGFR mutants at this time. Under these conditions, the damage introduced to control embryos was limited to the vicinity of the laser-illuminated area, and embryogenesis proceeded (*n*=15 embryos, Fig. 6E, Movie 7). When the EGFR mutant was wounded, the damage expanded to the neighboring region, separate from the epithelial disintegration initiated from the head region. The initial epidermal disintegration phenotype was limited to the side of laser wounding and took more than 30 min to initiate (Fig. 6F). After 90 min, the treated embryos collapsed ectopically in the thorax-abdominal regions (*n*=10 embryos, Fig. 6F, Movie 8). Cross-sectional views showed that wounding caused ectopic detachment of the epithelia from the vitelline membrane around the vicinity of the wounding region (Fig. S3A,B). In both control and EGFR-mutant embryos, ectopic detachment persisted for more than 1 h (Fig. S3C-F).

## DISCUSSION

In this study, we sought to understand the mechanisms that protect the epithelia from a variety of stresses present in developing embryos. By searching for conditions that exacerbate the apoptotic cell extrusion phenotype of EGFR mutants, we found that epithelial invagination severely enhances the phenotype. Invaginating epithelial cells create anisotropic contractile forces to induce the folding of the epithelia (Chung et al., 2017; Letizia et al., 2011; Nishimura et al., 2007; Sanchez-Corrales et al., 2018), which applies mechanical stress to the adherens junction. We found that EGFR-ERK signaling was upregulated, under the control of *rhomboid*, at the site where the apical cell membrane detached from the vitelline membrane. We conclude that the epithelium is mechanically supported by contact with the vitelline membrane; the activation of EGFR-ERK signaling, concomitant with the loss of contact, suppresses transient instability through the enforcement of adherens junctions. The functional overlap of the vitelline membrane and EGFR signaling was verified by the synergistic effect of removing both the vitelline membrane and EGFR signaling. Loss of EGFR function may reduce the threshold required for epithelial cells to tolerate instability from various sources. Here, we discuss the role of EGFR in stabilizing epithelia and how the vitelline membrane increases the robustness of morphogenetic movement.

### EGFR promotes cell surface contractility and prevents apical cell extrusion

*Drosophila* EGFR has multifaceted roles, including the regulation of patterned gene expression, cell survival and control of myosin contractility (Bergmann et al., 2002; Crossman et al., 2018; Hayashi and Ogura, 2020; Ogura et al., 2018; Price et al., 1989; Raz and Shilo, 1993; Schejter and Shilo, 1989). The latter two functions are relevant to this study. Myosin contractility and cell migration in cultured epithelial cells are controlled by the propagation of ERK activity through the activation of receptor tyrosine kinases (Aikin et al., 2020; Aoki et al., 2017), and the propagating wave of ERK has been observed in the mouse epidermis (Hiratsuka et al., 2015). In *Drosophila* epithelia, myosin accumulates at the adherens junction level to form cables spanning multiple cells, and functions to constrict junction length. Simultaneous imaging of myosin and ERK-FRET probes showed that ERK activation precedes myosin cable formation (Ogura et al., 2018; Fig. 6). These myosin cables apply contractile forces to the apical side of the epithelium to promote tissue invagination (Kondo and Hayashi, 2013; Nishimura et al., 2007; Ogura et al., 2018). A recent study on *Drosophila* pupal notum reported that sporadic cell death triggers transient EGFR-ERK activity in the surrounding cells that promptly seals the gap produced by extrusion of dead cells and repairs epithelial integrity (Valon et al., 2021). Concentrating myosin near the adherens junction ensures contractile tension is high in the apical surface so that the actomyosin cables function as a mechanical barrier to prevent cell extrusion to the apical side.

Rhomboid family proteins are the major activator of the EFGR ligand Spitz through intramembrane cleavage of the precursor form of Spitz in the Golgi apparatus (Shilo, 2016). The transcription patterns of *rhomboid* closely correlated with the patterns of EGFR-dependent ERK activity. We showed that *rhomboid* is a major inducer of ERK activity in embryos. The epithelial phenotype of *rhomboid* is mild compared with the extensive cell deaths and tissue disintegration in EGFR mutants, indicating that additional stimuli for EGFR must exist. It is unlikely that the other members of the seven rhomboid family genes fulfill that role because other members are either not expressed abundantly in the embryos or are non-proteolytic (Shilo, 2016). A potential alternative path for EGFR activation in the neighborhood of dying cells is stretch-induced EGFR activation, (Hino et al., 2020; Valon et al., 2021), in which apoptotic cells autonomously shrink and stretch to their neighbors, and activate the EGFR-ERK pathway that stimulates contractile myosin cables in a pattern surrounding the dying cells. Such a myosin cable was observed, shortly after ERK activation, in the apical cell junction in the area of laser-induced cell killing (Fig. 6D), which undergoes basalward movement and detachment from the vitelline membrane (Fig. S3), suggesting that stretch-induced EGFR-ERK-myosin activation prevents apical cell extrusion in the embryo. In EGFR mutants, the loss of contractility would cause a failure in integrity at the adherens junction, which was observed as gaps in E-cadherin localization (Fig. S2D).

### Vitelline membrane stabilizes epithelia

Apoptotic cells are extruded in either an apical or basal direction from cultured epithelium or zebrafish tissues (Eisenhoffer et al., 2012; Gu and Rosenblatt, 2012; Ohsawa et al., 2018; Tada, 2021). Basal cell extrusion is a common form of cell elimination in *Drosophila* imaginal discs (Ohsawa et al., 2018), and forced extrusion of polarity-deficient cells to the apical side causes luminal tumors (Vaughen and Igaki, 2016). In *Drosophila* embryonic epithelia, naturally or experimentally occurring apoptotic cells are mostly extruded to the basal side (Abrams et al., 1993, Fig. 2A,B). Although the basement membrane is a potential cue for basalward cell extrusion in imaginal discs, the accumulation of basement membrane components is not complete until stage 16 or later (Matsubayashi et al., 2017, 2020). This implies that another positional cue may restrict the direction of cell extrusion in the embryo. Close apposition of the apical cell membrane under pressure from the internal part of the embryo to the vitelline membrane serves as a physical barrier that restricts apical cell extrusion. In addition, part of the ectoderm adjacent to the hindgut invagination site has been shown to be connected to the vitelline membrane via integrin adhesion (Münster et al., 2019). This adhesion system may offer a more robust stabilization mechanism.

### Potential contribution of mitosis to tissue instability

We showed that clustered apoptosis accompanied the initial apical cell extrusion observed in EGFR mutants (Fig. 3). However, other clusters of apoptosis in the head and thoracic segments, which were tightly associated with the vitelline membrane, did not exhibit the apical cell extrusion phenotype (Fig. 4F,G). The notable exception was the apoptotic cell cluster in salivary gland invaginations (Fig. 4F). This implies that a cluster of apoptosis is not sufficient to cause apical cell extrusion, and additional conditions, other than invagination, must cooperate with the EGFR mutation in the head region. We showed that clusters with high mitotic activity were present in the head (Fig. S2A,B). These mitotic cells were present in the region of clusters of apoptotic cells in EGFR mutants (Fig. 3C-F), whereas the apically extruded cells did not contain mitotic cells (Fig. S2B). Mitotic cells undergo cell rounding, in which actin and myosin are redistributed to the cell cortex to increase cortical tension (Kunda et al., 2008; Stewart et al., 2011) and maintain adhesion to their neighboring cells through the equatorial E-cadherin belt. Rounded mitotic cells bulge out of the apical surface to cause detachment from the vitelline membrane (Kondo and Hayashi, 2013, Fig. S1I) and to apply stress to the weakened adherens cell junction, which is depleted of actin and myosin (Ragkousi and Gibson, 2014). We speculate that clustered mitotic activity in healthy cells in the head of EGFR mutants causes detachment from the vitelline membrane and enhances instability in the epithelia, leading to apical cell extrusion.

### Concluding remarks

It should be noted that embryonic development in many experimental model organisms, such as mice, chicks and zebrafish, can proceed outside egg membranes, although the contribution of the mouse zona pellucida to embryo polarization has been documented (Kurotaki et al., 2007). Presumably, the relatively slow developmental time of these embryos (more than threefold slower than *Drosophila*) would buffer the negative impact of mitosis and invagination on epithelial stability and allow enough time for tissues to repair any damage that might occur during morphogenesis, making embryonic membranes dispensable. In addition, two cell adhesion systems, the septate junction mediating adhesion of lateral cell membranes and the integrin-basement membrane adhesion systems, develop only in the late embryonic stages (stage 15 or later, Tepass and Hartenstein, 1994), leaving the task of maintaining epithelial cell adhesion during early morphogenesis mostly to the adherens junction. The simplicity of the adhesion system may allow flexibility in coping with rapid morphogenesis at the expense of an increased risk of tissue damage. The recruitment of the vitelline membrane as a supporting medium and EGFR signaling as a tissue repair mechanism permits rapid and robust embryonic development of *Drosophila*, which is advantageous in a varying reproductive environment.

## MATERIALS AND METHODS

### Experimental models

All *Drosophila* strains (Table S1) were cultured in standard yeast-cornmeal media at 25°C. Embryos were staged according to morphological criteria and time course (Campos-Ortega and Hartenstein, 1997).

### Immunostaining

Embryos were collected on apple juice-2% agar plates covered with yeast. Embryos were dechorionated with 100% household chlorine bleach for 2 min and fixed for 40 min with a 1:1 mixture of 4% w/v paraformaldehyde in PBS and 100% heptane at room temperature. The embryos were devitellinized by replacing the water phase with 100% methanol with vigorous shaking. After removing the heptane and washing with 100% methanol, the embryos were rehydrated with blocking buffer [2% bovine serum albumin, 0.1% Triton X-100, 0.1% polyoxyethylene (20) sorbitan monolaurate in PBS] for 30 min. The embryos were stained with primary antibodies at 4°C overnight. After five washes in blocking buffer, the embryos were stained with secondary antibodies at 4°C overnight. The source and working concentrations of primary and secondary antibodies and reagents are listed in Table S1. Immunostained embryos were mounted in Vectashield and observed under a confocal microscope (Olympus FV1000) using a UPlanSApo 20× NA 0.75 with a 1.5 zoom, and a UPLanSApO 60× water NA 1.2. Each image was acquired as an 800×600 pixel image with an optically optimum *z*-interval.

### Live imaging

Dechorionated *Drosophila* embryos were mounted on a glass-bottom med dish (Iwaki) coated with glue and extracted with heptane using double-sided sticky tape (Scotch). Each confocal image was acquired using a laser-scanning confocal microscope (FV1000, Olympus) with the following lenses: PlanApo N 60×NA1.42 oil (most of the live imaging), UPlanSApo 60×NA1.30 silicon (for FRET imaging with fluorescent dextran injection in Fig. 1F,G), UPlanSApo 30× NA1.05 silicon (for observation of the whole embryo), and PlanApo 60× NA1.40 oil IR lens (for IR irradiation). Each image was acquired as a 640×480-pixel image with an optimum *z*-interval, every 3 or 5 min over 5 h with GaAsP detectors. The images were de-noised and projected using ImageJ-Fiji software. Förster resonance energy transfer imaging and data processing were performed as described previously (FRETratioFx; Table S1; Ogura et al., 2018, 2019).

### Fluorescence dextran injection

Dechorionated embryos at stages 11-12 were mounted on a cover glass (Matsunami) and covered with silicone oil (Shinetsu silicone). A solution of tetramethyl rhodamine-conjugated dextran, 3000 MW (1 µg/µl), was diluted in PBS and injected into the perivitelline space of the embryos. The glass needles were prepared by pulling a glass capillary with a needle puller (Sutter).

### Embryo dissection and culture

Eggs were collected overnight at 18°C, and stage 11-12 embryos were selected from the dechorionated embryos. They were placed on a polystyrene tissue culture dish (Iwaki) and covered with Schneider's *Drosophila* medium supplemented with 10% fetal bovine serum and antibiotics (penicillin and streptomycin). The vitelline membrane was punctured by pricking at the anterior end of the embryo with a stainless steel needle (Shiga Konchu), and half of the disrupted embryos were pushed out of the vitelline membrane. Tissue fragments were collected without vitelline membrane from at least 10 embryos in each experiment and placed in poly-L-lysine-coated glass-bottomed dishes (Matsunami, D141410) for confocal imaging. Epidermal tissue fragments facing the apical side towards the glass were imaged within 1 h of dissection. A list of reagents used is provided in Table S1.

### Laser-induced wounding

The IR-LEGO system (IR-LEGO-1000, Sigma-Koki) was combined with a confocal microscope (FV1000, Olympus) equipped with GaAsP detectors. The IR laser was introduced through the lateral camera port of an inverted microscope (IX81, Olympus). The laser intensity was adjusted to 100 mW or 150 mW to produce local wounds (Kamei et al., 2009; Kato et al., 2016; Miao and Hayashi, 2015). Time-lapse confocal images were captured immediately after IR laser application.

## Supplementary Material

10.1242/develop.201231_sup1 Supplementary information
