# Supplementary material for: Epidermal growth factor receptor signaling protects epithelia from morphogenetic instability and tissue damage in Drosophila
Source: Development. 2023 Mar 9;150(5):dev201231. doi: 10.1242/dev.201231 (PMC10108703; doi:10.1242/dev.201231)
Supplement: Supplementary information [file develop-150-201231-s1.pdf]

Supplementary Figure S1.

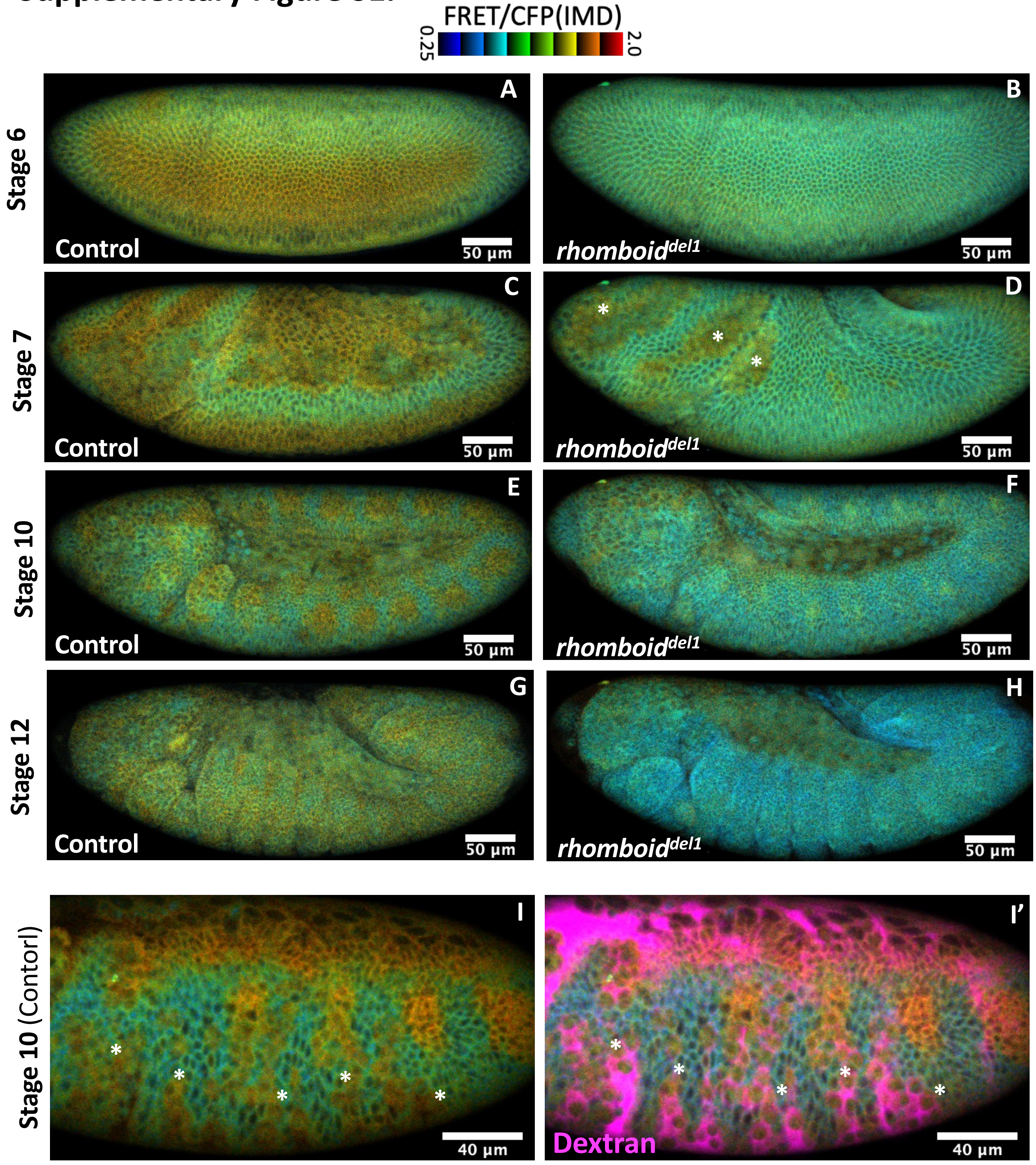

Fig. S1. ERK-FRET images during embryogenesis. Control embryos (A,C,E,G). Rhomboid mutant (B,D,F,H) embryos. The asterisk indicates a mitotic cell cluster with high FRET signal reflecting the CDK1 activity independent of rhomboid. (D). Asterisks indicate mitotic cells with FRET activity induced by Cdk1. I, I'. ERK-FRET image (I) and an overlay of perivitelline dye (I') in stage 10 embryos. Asterisks indicate mitotic cell clusters that detach from the vitelline membrane.

Supplementary Figure S2.

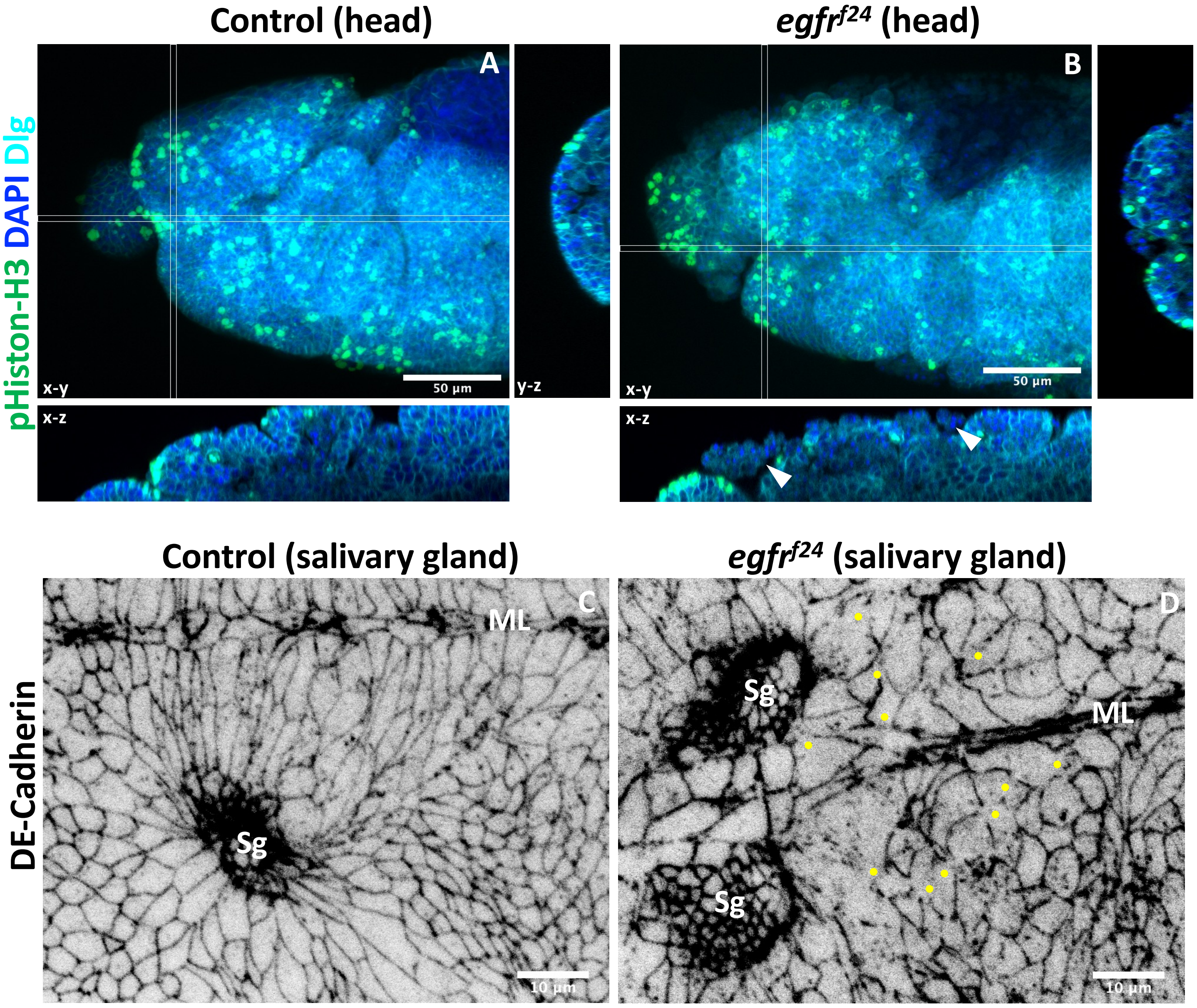

Fig. S2. Patterns of mitotic cells and E-cadherin localization.

A, B. Mitotic cell marker phosphorylated histone H3 staining of control (A) and EGFR mutant (B) cells in the head. Closed arrowhead indicate the cluster of apically extruded cells. C, D. E-cadherin localization around the invagination site of the salivary gland. Control embryo (C). EGFR mutant embryo (D). Yellow dot shows the gaps of the junctional E-cadherin localization. N=4 for each genotype. ML: ventral midline.

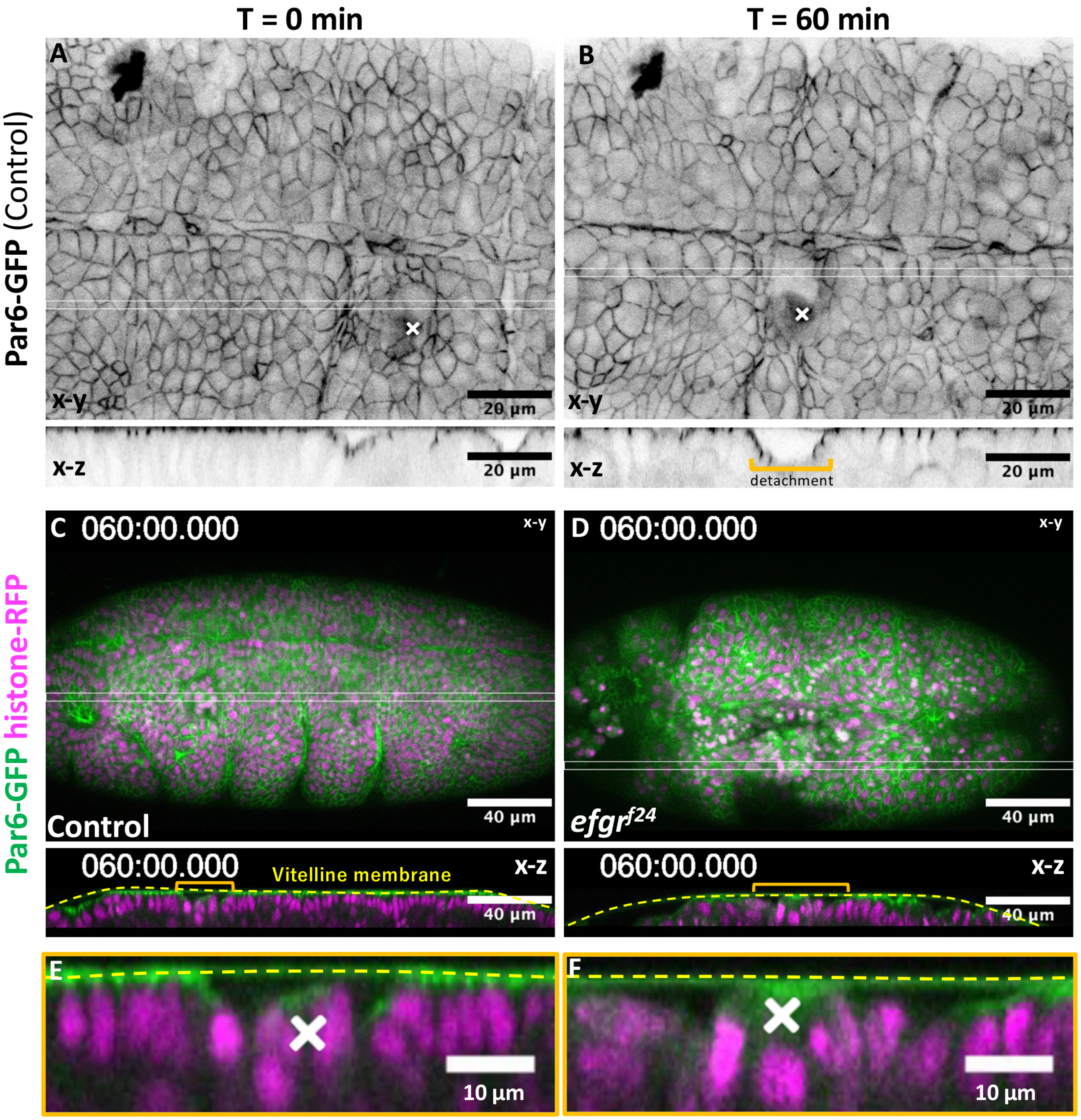

Fig. S3. Detachment of epidermis from the vitelline membrane by wounding.
A, B. X-Y and X-Z sections of the region near the wound site. The x-z section of the double horizontal line is shown. A. Immediately after laser wounding. The cells were attached to the vitelline membrane. B. 60 min later, a large gap appeared. The gap was observed 5 min after wounding and gradually increased in size. C-F. Cross-sectional views of wounded regions of the control (C, E) and EGFR mutant (D, F) 60 min after wounding. Note in enlarged views (E, F) that apical Par6-GFP was maintained in the control (E), but lost in the mutant (F). Related to Fig.s 6 E, F, and movie S7. Cross marks; wounded site.

Table S1. Key resource table

Click here to download Table S1

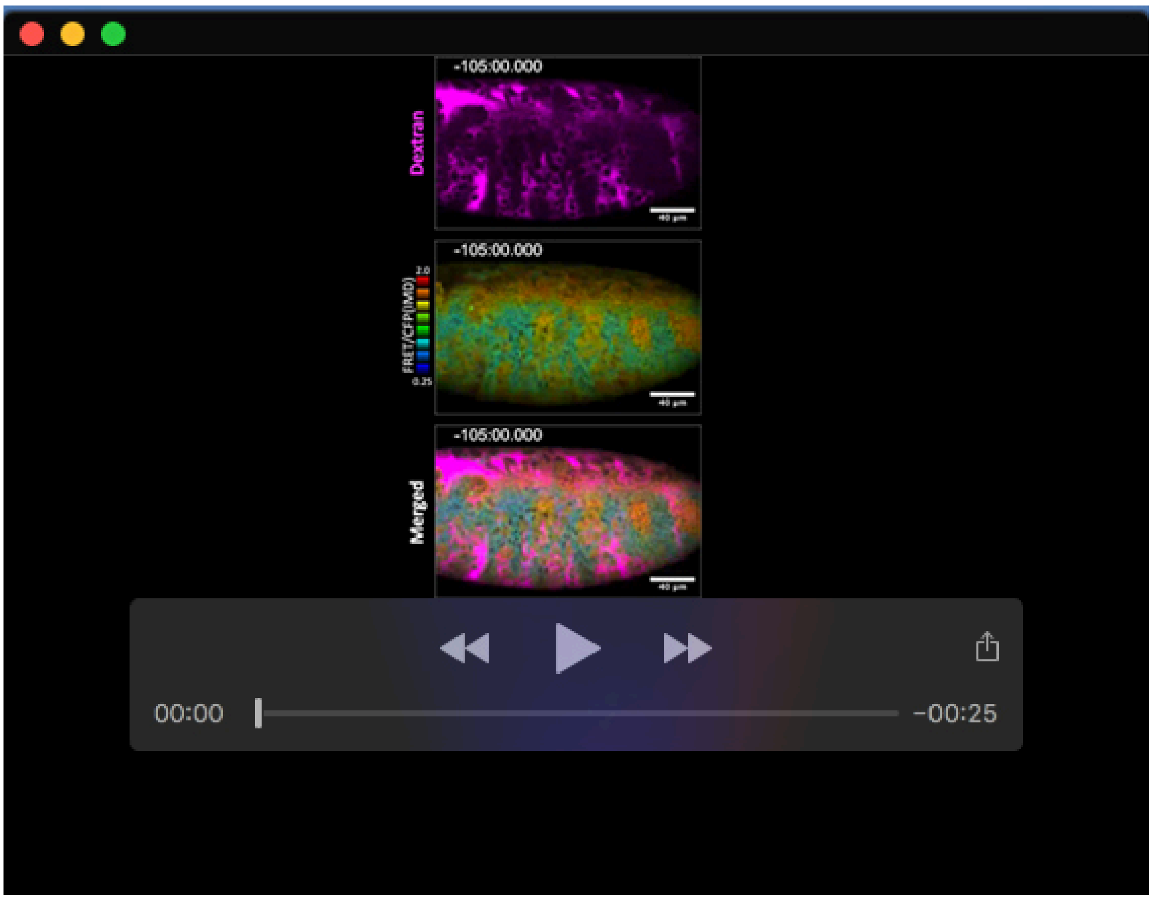

Movie 1. ERK-FRET imaging of embryos injected with a fluorescent tracer (rhodamine dextran) in the perivitelline space. Related to Fig. 1D-G. Time (t) = 0 was set at the time of tracheal invagination of T1 segment.

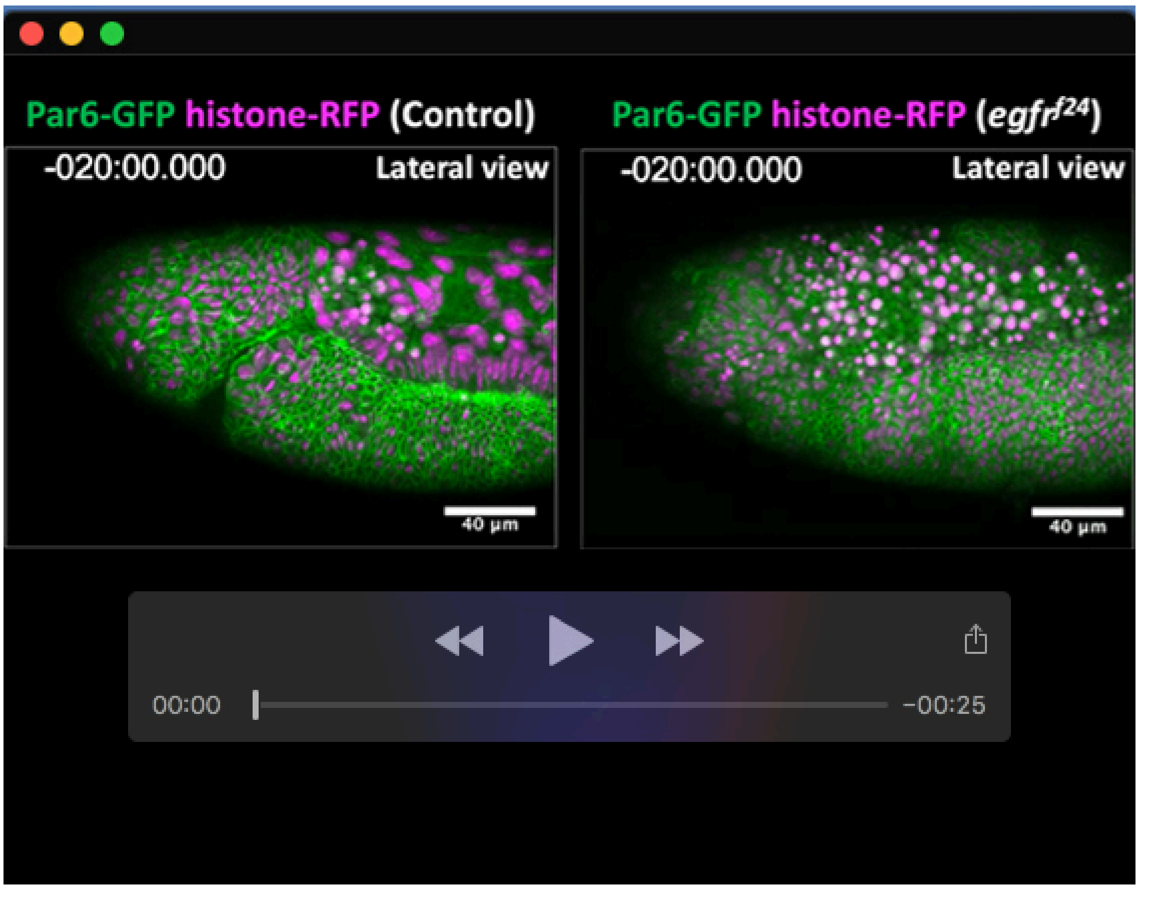

Movie 2. Tissue collapse phenotype of the EGFR mutant. Imaging was started in the late stage 10. Time t = 0 was set at the time of full opening of the tracheal pit at T2. Related to Fig. 2D, E.

Development • Supplementary information

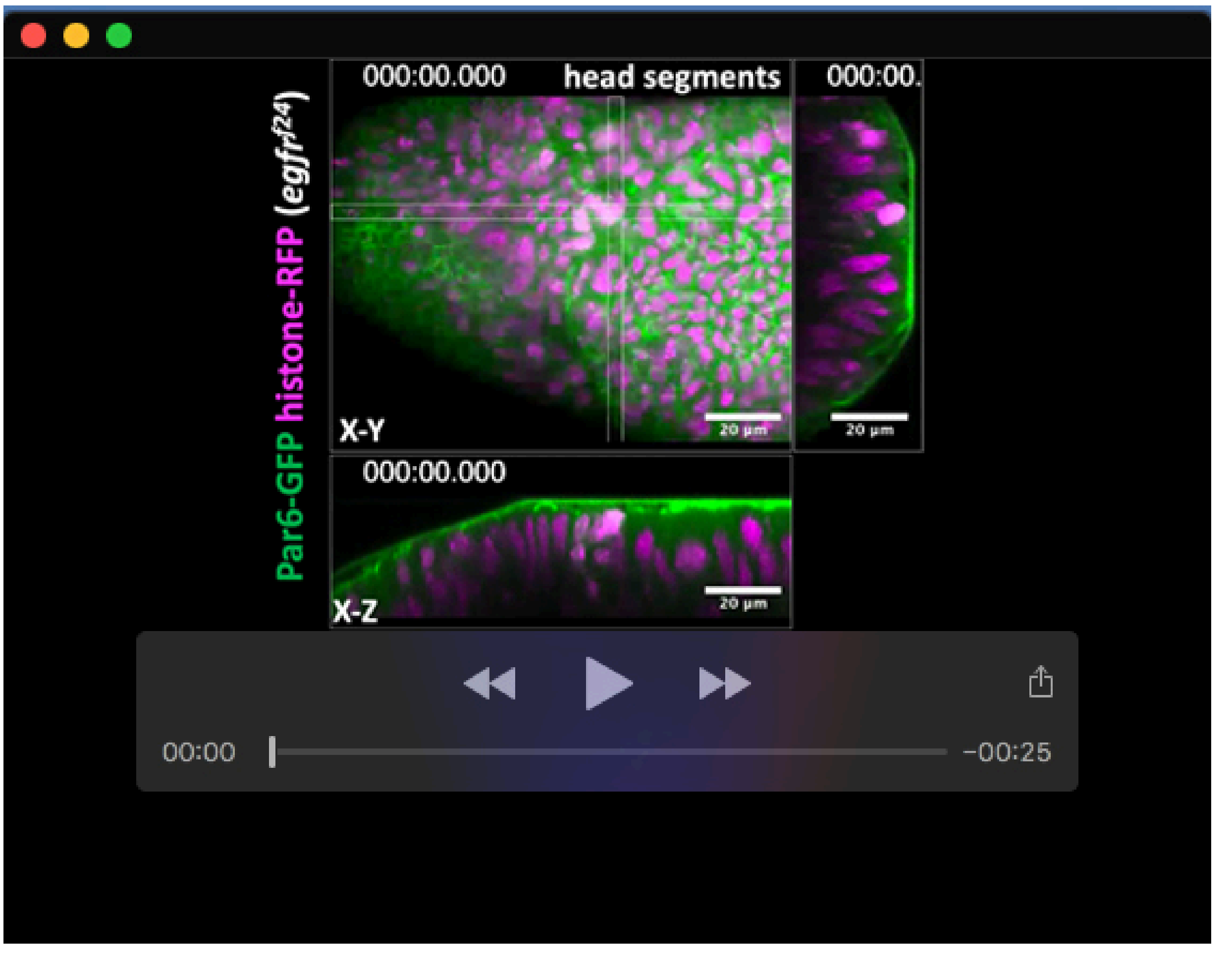

Movie 3. Apical cell extrusion in the EGFR mutant head. The time to the first apically extruded cell was set as t = 0. This cell was tracked by adjusting the positions of the x-z and y-z slices. Related to Fig. 3G.

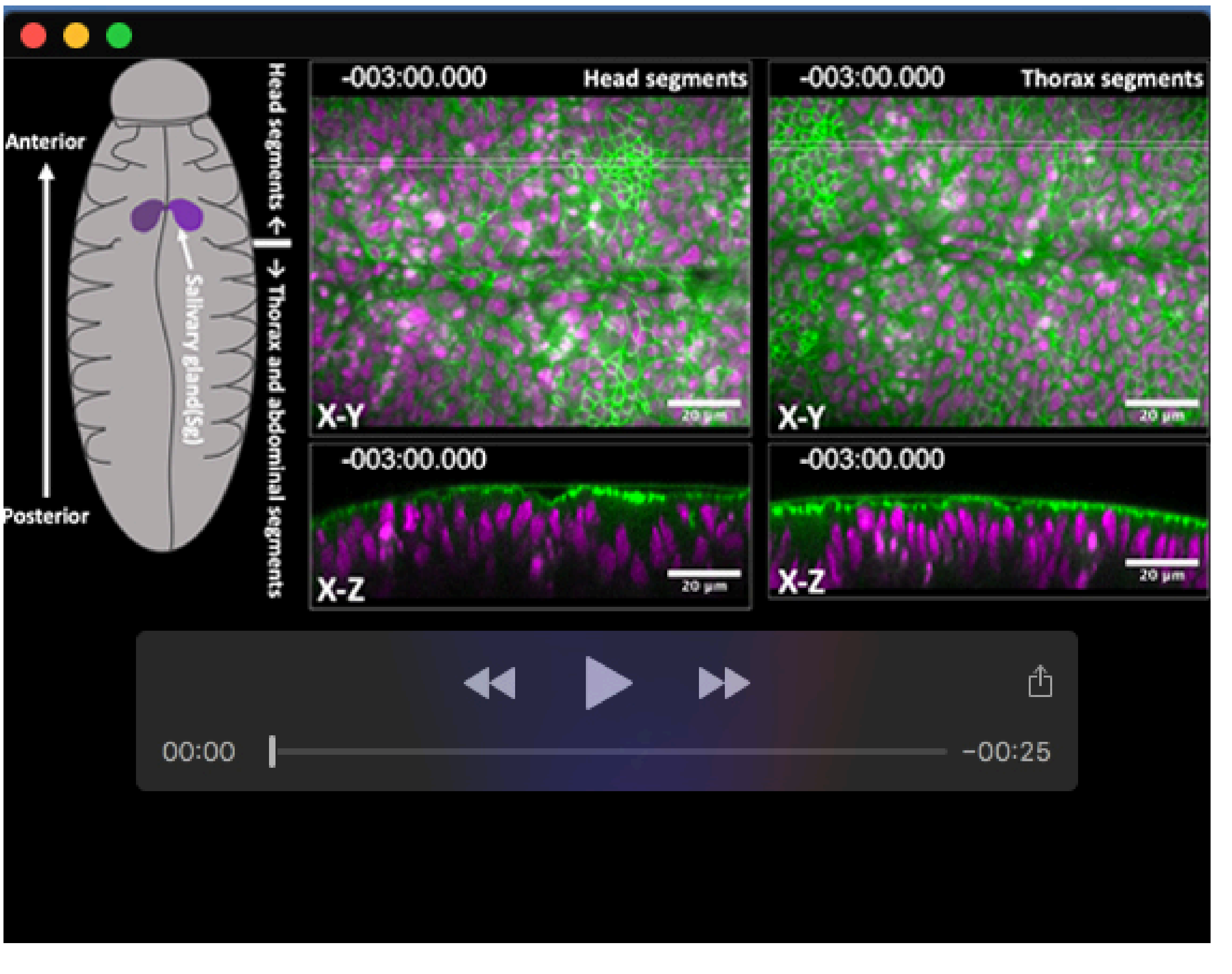

Movie 4. Apical cell extrusion at the salivary gland invagination site of EGFR mutants. Ventral views of the head and thoracic regions of the two embryos were obtained. Time t = 0 was set at the beginning of invagination. Related to Fig. 4F, G.

Development • Supplementary information

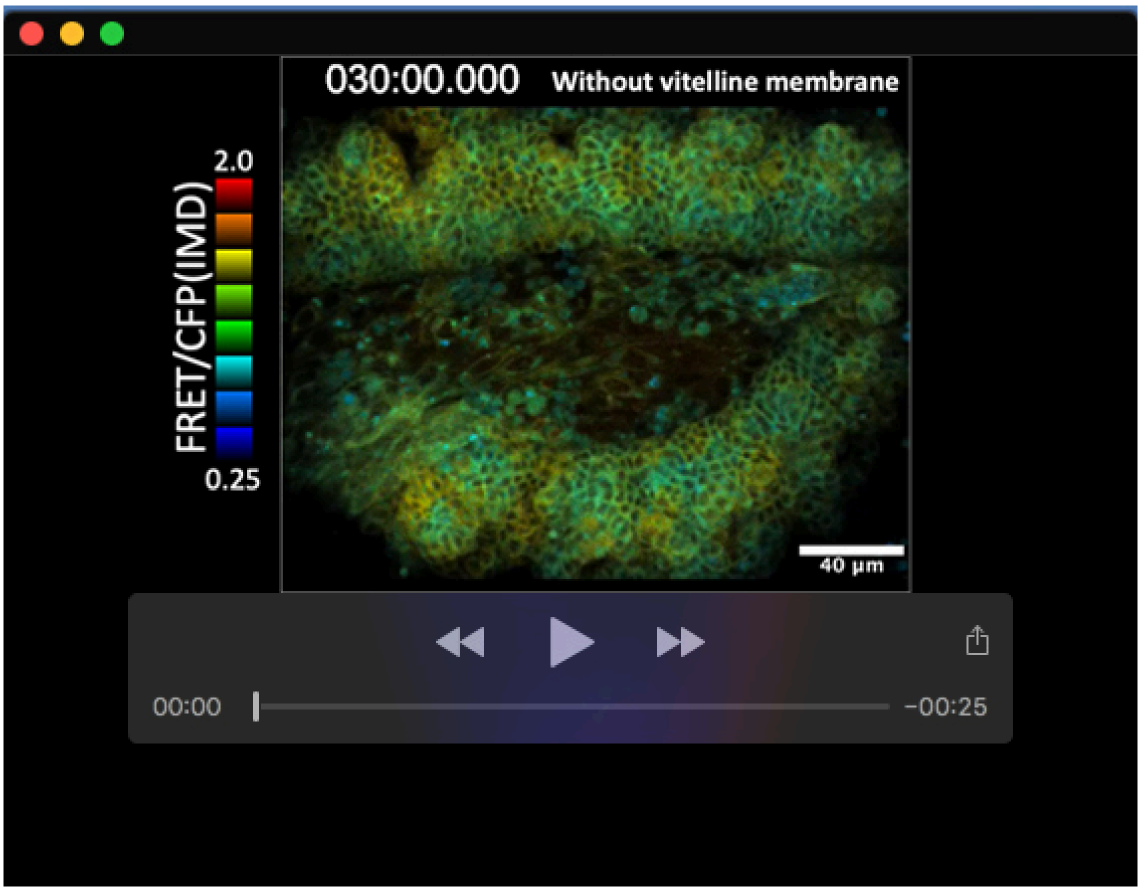

Movie 5. ERK FRET activity in a tissue explant from stage 11 embryo. The FRET activity in the tracheal invagination site was maintained after devitellinization. Related to Fig. 5E.

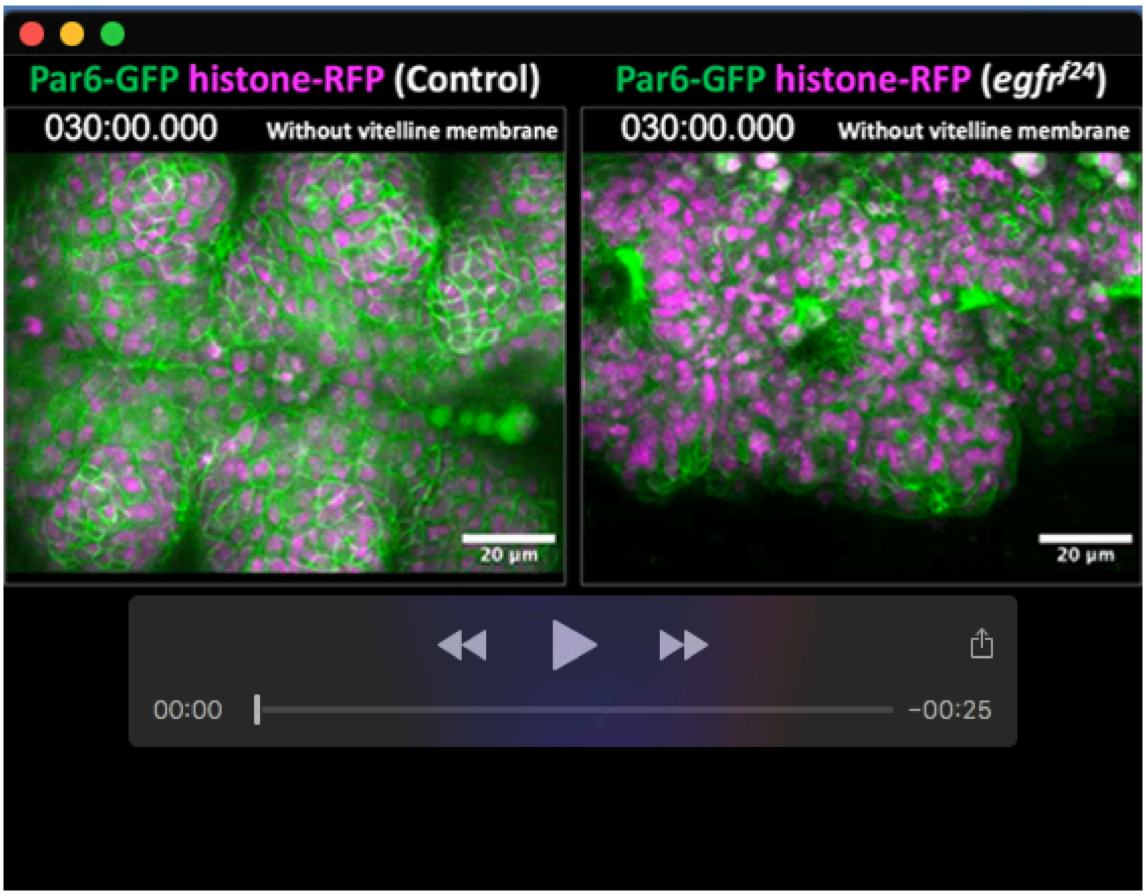

Movie 6. Tissue explant experiment. Tissue fragments of the control and EGFR mutant, removed from the vitelline membrane, and cultured *in vitro*, were imaged. Related to Fig. 5G.

Development • Supplementary information

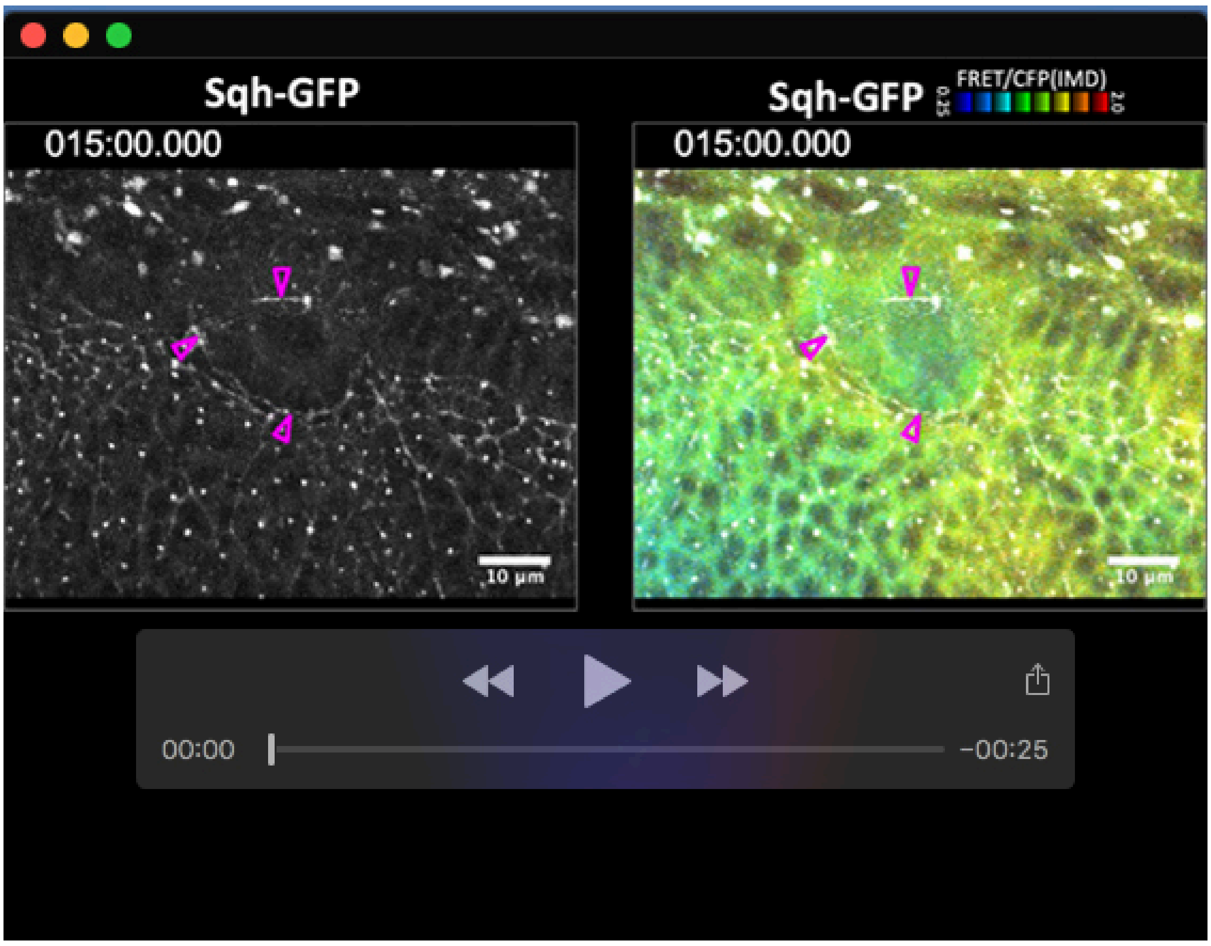

Movie 7. Responses of myosin (myosin light chain GFP) and ERK signaling (ERK-FRET) to laser-induced wounding in control embryos. Related to Fig. 6D.

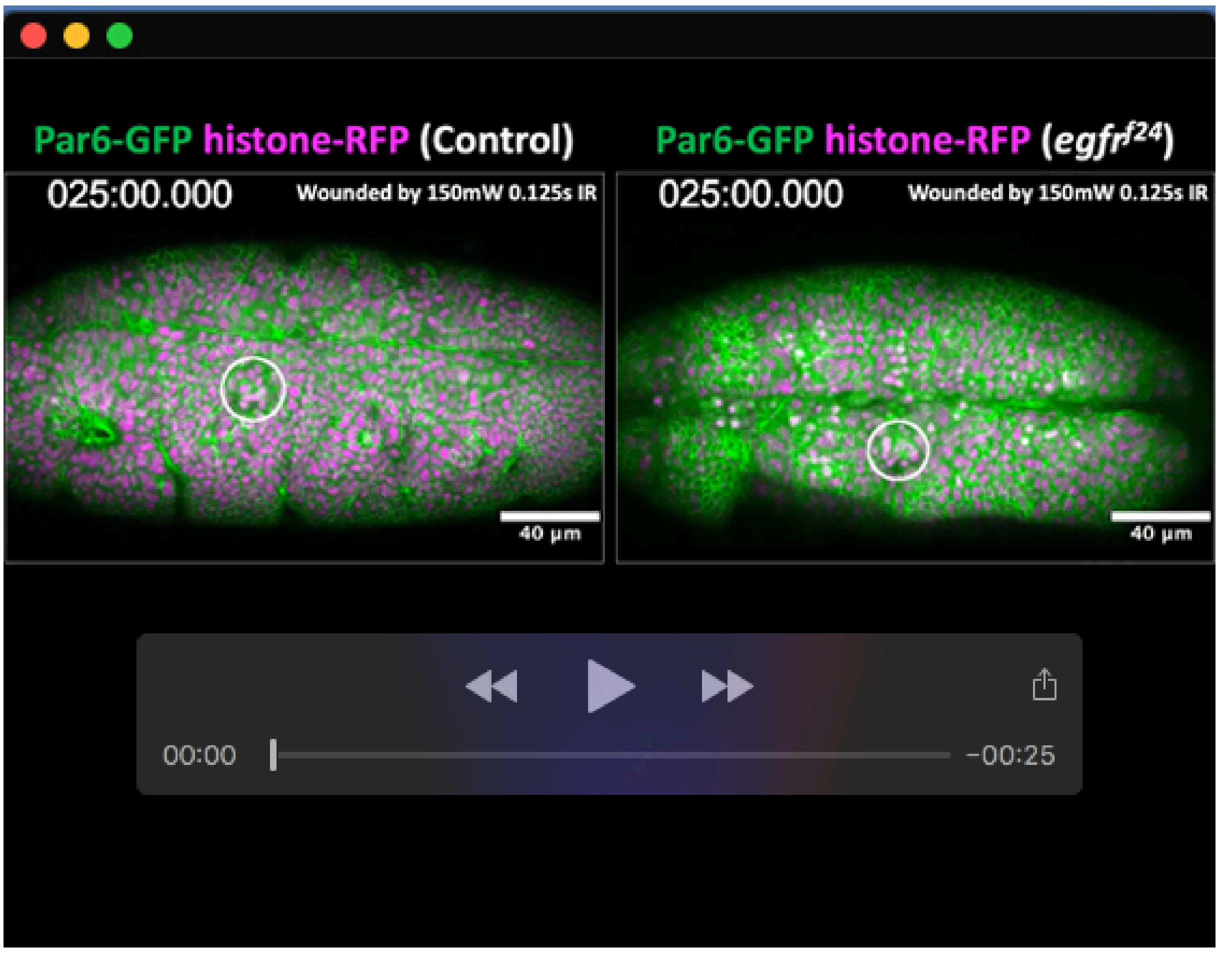

Movie 8. Laser-induced wounding in EGFR-mutant embryos. Embryos at stage 12 prior to tissue collapse in the thorax were chosen for the experiment. Related to Fig. 6E, F.

Development • Supplementary information

References

Bardet, P.-L., Kolahgar, G., Mynett, A., Miguel-Aliaga, I., Briscoe, J., Meier, P. and Vincent, J.-P. (2008). A fluorescent reporter of caspase activity for live imaging. *Proc. Natl. Acad. Sci. USA* **105**, 13901-13905. doi:10.1073/pnas.0806983105

Bier, E., Jan, L. Y., & Jan, Y. N. (1990). rhomboid, a gene required for dorsoventral axis establishment and peripheral nervous system development in Drosophila melanogaster. *Genes Dev*, **4**, 190–203. doi: 10.1101/gad.4.2.190

Clifford, R. J. and Schü pbach, T. (1989). Coordinately and differentially mutable activities of torpedo, the Drosophila melanogaster homolog of the vertebrate EGF receptor gene. *Genetics* **123**, 771-787. doi:10.1093/genetics/123.4.771

Kondo, T. and Hayashi, S. (2013). Mitotic cell rounding accelerates epithelial invagination. *Nature* **494**, 125-129. doi:10.1038/nature11792

Miao, G. and Hayashi, S. (2015). Manipulation of gene expression by infrared laser heat shock and its application to the study of tracheal development in Drosophila. *Dev. Dyn.* **244**, 479-487. doi:10.1002/dvdy.24192

Ogura, Y., Wen, F.-L., Sami, M. M., Shibata, T. and Hayashi, S. (2018). A switch-like activation relay of EGFR-ERK signaling regulates a wave of cellular contractility for epithelial invagination. *Dev. Cell* **46**, 162-172.e5. doi:10.1016/j.devcel.2018.06.004

Ogura, Y., Sami, M. M., Wada, H. and Hayashi, S. (2019). Automated FRET quantification shows distinct subcellular ERK activation kinetics in response to graded EGFR signaling in Drosophila. *Genes Cells* **24**, 297-306. doi:10.1111/gtc.12679

Uemura, T., Oda, H., Kraut, R., Hayashi, S., Kotaoka, Y. and Takeichi, M. (1996).Zygotic Drosophila E-cadherin expression is required for processes of dynamic epithelial cell rearrangement in the Drosophila embryo. *Genes Dev*. **10**, 659-671. doi:10.1101/gad.10.6.659

White, K., Grether, M. E., Abrams, J. M., Young, L., Farrell, K., & Steller, H. (1994). Genetic Control of Programmed Cell Death in Drosophila. *Science*, **264,** 677–683.doi: 10.1126/science.8171319

Wirtz-Peitz, F., Nishimura, T. and Knoblich, J. A. (2008). Linking cell cycle to asymmetric division: aurora-a phosphorylates the par complex to regulate numb localization. *Cell* **135**, 161-173. doi:10.1016/j.cell.2008.07.049

Development • Supplementary information
