# Supplementary material for: Epidermal growth factor receptor signaling protects epithelia from morphogenetic instability and tissue damage in Drosophila
Source: Development. 2023 Mar 9;150(5):dev201231. doi: 10.1242/dev.201231 (PMC10108703; doi:10.1242/dev.201231)
Supplement: Supplementary file 1 [file develop-150-201231_review_history.pdf]

Epidermal growth factor receptor signaling protects epithelia from morphogenetic instability and tissue damage in *Drosophila*

Kentaro Yoshida and Shigeo Hayashi
DOI: 10.1242/dev.201231

Editor: Thomas Lecuit

Review timeline
Original submission: 30 August 2022
Editorial decision: 31 October 2022
First revision received: 4 January 2023
Accepted: 9 February 2023

Original submission

First decision letter

MS ID#: DEVELOP/2022/201231

MS TITLE: Epidermal growth factor receptor signaling protects epithelia from morphogenetic instability and tissue damage in Drosophila

AUTHORS: Kentaro Yoshida and Shigeo Hayashi

I apologize for the long delay before being to come back to you. I have now received all the referees' reports on the above manuscript, and have reached a decision. The referees' comments are appended below, or you can access them online: please go to BenchPressand click on the 'Manuscripts with Decisions' queue in the Author Area.

As you will see, the referees express considerable interest in your work, but have some significant criticisms and recommend a revision of your manuscript before we can consider publication. If you are able to revise the manuscript along the lines suggested, which may involve further experiments, I will be happy receive a revised version of the manuscript. Your revised paper will be re-reviewed by one or more of the original referees, and acceptance of your manuscript will depend on your addressing satisfactorily the reviewers' major concerns. Please also note that Development will normally permit only one round of major revision. If it would be helpful, you are welcome to contact us to discuss your revision in greater detail. Please send us a point-by-point response indicating your plans for addressing the referee's comments, and we will look over this and provide further guidance.

Please attend to all of the reviewers' comments and ensure that you clearly highlight all changes made in the revised manuscript. Please avoid using 'Tracked changes' in Word files as these are lost in PDF conversion. I should be grateful if you would also provide a point-by-point response detailing how you have dealt with the points raised by the reviewers in the 'Response to Reviewers' box. If you do not agree with any of their criticisms or suggestions please explain clearly why this is so.

Reviewer 1

Advance summary and potential significance to field

In this manuscript, Yoshida and Hayashi describe in a very interesting epithelial phenotype associated with EGFR depletion in Drosophila embryo. Using a combination of genetics, FRET

© 2023. Published by The Company of Biologists under the terms of the Creative Commons Attribution License (https://creativecommons.org/licenses/by/4.0/).

imaging and live imaging they found that EGFR depletion promotes epithelial collapse in the Drosophila embryo, especially in regions undergoing invagination and detachment from the vitelin membrane. By directly removing the vitelin membrane, they further suggest that EGFR mutant cells are more sensitive to vitelin membrane detachment which provokes massive apoptosis. Epithelial defects not only emerge in regions of invagination, but are also associated with clustered extrusion which depending on the regions can either occur apically (which is atypical in Drosophila, except in the adult midgut) or basally.

Overall, many observations are very interesting and open a lot of questions about the relationship between vitelin membrane, cell survival,extrusion epithelial stability and EGFR. There is however very little mechanistic insights at this stage and most of the putative explanations for the epithelial stability phenotype remain quite hypothetical. I do believe that the fine phenomenology and precise characterization of the phenotype justify a publication by itself but few genetic experiments may help to clarify/sort some of the explanations proposed by the authors and would make the manuscript more compelling.

Comments for the author

1. Three main non-exclusive mechanisms could explain epithelial collapse upon EGFR depletion :

A. High rate of apoptosis and clustered cell death. The high rates of apoptosis may prevent proper execution of extrusion which could lead to sealing defects and catastrophic tissue collapse
B. A deregulation of tension : high tissular tension may promote cell detachment and tissue collapse
C. A deregulation of adhesive forces : a lowering of cell-cell adhesion and/or adhesion to vitelin membrane may weaken cell bound and promotes tissue collapse

Many of these parameters are quite interconnected but some key experiments could be performed to partially sort their contribution:

A. Blocking caspase/apoptosis in the contact of EGFR depletion : could the author overexpress Diap1 or p35, or use the deletion H99 to lower down apoptosis in EGFR mutant background and test if this affect the extrusion events and delay epithelial collapse ? If the genetic is too complicated, the authors may use injection of the pan caspase inhibitor zvad-fmk or even use genetic inhibition of apoptosis combined a drug inhibition of EGFR/ERK provided it phenocopy EGFR null (e.g.: trametinib, a MEK inhibitor, works potently in flies).

B,C. Could the authors provide some comparison of the levels of E-cad and MyoII in the EGFR mutant compared to the control embyos, specially in the region that will collapse first ?

B,C. Is there any evidence of genetic interactions between E-cad, and/or MyoII/Rho1 and EGFR ? (enhancement or partial rescue of the phenotype upon modulation of E-cad, or MyoII activity)

2. So far, the authors have shown that vitelin membrane detachment promotes epithelial collapse especially in absence of EGFR. However, it is not clear at this stage whether ERK activation pattern in the WT is related causally to the local invagination and whether vitelin membrane detachment does promote ERK. Actually, movie S1 clearly shows many regions where ERK activation seems to precede tissue invagination and detachment. While it could fit with the notion that EGFR/ERK is required to protect zones of detachment, I would recommend to remain more cautious on the causal link between vitelin membrane detachment and EGFR/ERK activation (as currently stated in the discussion). Unless the authors can show an increase of ERK activation upon vitelin membrane detachement like in Fig 5, I would amend the text whenever this point is mentioned.
Could the author also try to evaluate more systematically whether ERK activation precede / is concomitant with / or comes after the detchament from the vitelin membrane ?
Minor points:

Minor pt 1: Page 2 : sentence : " promotes de closure of the epithelial opening after apoptotic extrusion (Moreno et al. 2019, Valon et al. 2021)". I am not sure this is what was shown in these articles (the first one shows that ERK can be modulated by mechanics and the second describes

© 2023. Published by The Company of Biologists under the terms of the Creative Commons Attribution License (https://creativecommons.org/licenses/by/4.0/).

pulses of ERK that change cell death distribution, but non-of them address the role of EGFR for closure and wound healing to my knowledge).

Minor pt 2: The first generation of EKAR can also be sensitive to Cdk1 (which could explain the activation seens during mitosis), see for instance https://www.life-science-alliance.org/content/5/1/e202101206, Lin et al Life science alliance 2021. So that could explain part parttern observed at stage 10 which colocalises with dextran (and has nothing to do with EGFR ERK). Indeed they are still here upon Rhomboid depletion. It is not an essential point for this study but it might be worth mentioning this potential caveat. Along this line, Rhomboid does not really seem to reduce the signal in the groove.

Minor pt 3 : In Figure 3F : It was not absolutly clear whether the particle shown with the yellow arrow are indeed Dcp1 negative. It sometime seems like a large cell contains subcompartment that are Dcp1 positive. Do the authors have more example of apical caspase negative cell specially isolated one that can be identified without ambiguity ?

Minor pt 4: Could the authors give a sense of the time required to observed tissue collapse upon vitellin membrane removal compared to the time to see collapse in EGFR mutant non-devitellinized embryo ? Is the collapse indeed occuring earlier upon vitelin membrane removal compared to EGFR mutant with vitelin membrane ?

Minor pt 5: It might be good to give more details in the methods on the genotypes. For instance, it was not clear to me whether the egfr f24 mutants were zygotic nulls or maternal/zygotic mutants. It might be worth giving this type of details for all the mutants used in the study.

Reviewer 2

Advance summary and potential significance to field

The manuscript from Yoshida and Hayashi describes a study of the involvement of the Drosophila EGF receptor (EGFR) in maintaining epithelial integrity during embryogenesis. EGFR, the most influential receptor mediating signaling via the MAP kinase (MAPK) pathway in Drosophila embryos, participates in a wide range of developmental processes. EGFR embryonic mutant phenotypes are therefore complex, and considerable effort is necessary in order to tease apart the different functional requirements. In this interesting paper, the authors use a range of experimental approaches in order to put forward the novel notion that the EGFR signaling pathway acts to restrict extrusion of (dead) cells to the basal side of epithelia during embryonic morphogenesis. A related notion is that this activity of EGFR is required and stimulated in regions (e.g segmental grooves) where the epidermal epithelium pulls away from the overlying vitelline membrane (VM).

Comments for the author

I would like to state at the outset that I encountered considerable difficulty in following the manuscript narrative, which struck me as unfocused and- at times- confusing, and I had to read portions of the text several times, in order to piece together the framework described above. Considerable reorganization is therefore necessary, in my opinion, in order to present the “story” in a substantially more coherent manner. My recommendation is that the authors clarify ahead of time where they are going, describe their work in a fashion that adheres and relates to this stated scenario, and contend better with questions that the data brings up. More specifically:

1. One issue is that the Results section starts off in unconvincing fashion, with an experiment in which a spatial correspondence is claimed between regions (e.g segmental grooves) showing both heightened ERK activity (as monitored with a FRET sensor) and separation between the embryo ectoderm and the vitelline membrane (observed by accumulation of injected Dextran). The authors then attempt to strengthen these rather indirect observations for EGFR function by showing that ERK activity is reduced in rhomboid-1 (rho-1) mutant embryos, which also display a somewhat abnormal measure of apical cell extrusion. This however, is a weak argument, given the generally mild disruption to morphogenesis observed in rho-1 (as well as in Spitz ligand) mutant embryos,

© 2023. Published by The Company of Biologists under the terms of the Creative Commons Attribution License (https://creativecommons.org/licenses/by/4.0/).

which, in turn, argues for an alternative means of EGFR activation in this context, an issue which the authors themselves raise in the Discussion.

I can appreciate the reasoning behind starting the manuscript in this fashion, but it really works against it. My strong recommendation would be to begin with the data and text around Figure 2, stating that the initial motivation for the study is- as already brought up by the authors- a desire to understand the severe morphogenesis phenotype of EGFR mutant embryos, which is not readily explained by currently established roles of this key receptor, and proceed from there.

2. The experiments described in Figures 3 and 4 appear to be put forward in order to establish that apical extrusion of cells which have undergone apoptosis, will occur when two conditions are met- epidermis/vitelline membrane (VM) separation and absence of EGFR activity. First off, this needs to be stated clearly. Second- what do the authors think happens in EGFR mutants that initiates and leads to disintegration of the epithelium? They claim to observe that “A gap between the apical cell surface vitelline membrane appeared”. Is the idea that this represents a stochastic event with dire consequences in EGFR mutants? Is it a normal event observed in wildtype embryos as well? Does it occur more frequently in the head region (so as to explain the anterior-to-posterior progress of disintegration in the mutants)?

3. EGFR is well known to function as a “survival factor” that limits apoptosis, and this activity has been shown to be consequential to Drosophila embryonic development (eg- Crossman et al 2018). To what degree does the excessive apoptosis seen in EGFR mutant embryos (and documented in this study as well) contribute to epithelial disintegration? Perhaps the system becomes overwhelmed by excessive cell death? The authors should consider an experiment in which apoptosis is blocked in the EGFR mutant background and assess the consequences and how they reflect on their model.

4. A couple of remarks regarding data presented in the figures:

-- As discussed above, the data presented in Figure 1 is problematic and not a good way to start the Results section. However, while the rhomboid experiments should, in my opinion, be removed altogether there is justification for presenting the wildtype FRET sensor and Dextran injection data at a later phase of the text, although not necessarily linked. In any case- if and when presented, the FRET sensor images should be accompanied by a matching “heatmap” schematic or the like, as the pattern of the signal is difficult to make out from the images.

-- When assessing the consequences of laser wounding (Figure6 E-F), a panel series should be added in which an age-matched unwounded EGFR mutant embryo (expressing Par6-GFP and Histone-RFP) is monitored, to allow comparison of the progress of development in the thorax/abdomen to the wounded embryo.

First revision

<u>Author response to reviewers' comments</u>

Summary of changes
1. Original Figure 1A-B (ERK FRET expression in control and rhomboid mutants) was moved to Supplementary Fig. S1 in a modified form.
2. ERK FRET and fluorescent dextran pictures showing mitotic cells activating the ERK FRET reporter and causing detachment of cells from the vitelline membrane were added as Supplementary Fig. S1I, I’.
3. Original Supplementary Fig. S1AB was removed because it provided redundant information.
4. Original Figure 1C (apical cell extrusion phenotype of *rhomboid* mutants) was removed as suggested by reviewer 2. This does not alter the conclusion of this paper.
5. Analysis of egfr; Df(3L)H99 double mutant embryos was added to revised Fig. 3. The data shows apoptosis is required for epithelial destabilization in EGFR mutants,
6. Images of E-cadherin distribution, showing adherens junction becomes discontinuous in EGFR mutants, were added as new Fig. S2C, D.

© 2023. Published by The Company of Biologists under the terms of the Creative Commons Attribution License (https://creativecommons.org/licenses/by/4.0/).

7. Movies of a dissected embryo showing the ERK-FRET pattern are maintained at least 60 minutes after devitellinization.
8. Text was modified to incorporate the suggestions from the reviewers (highlighted with a yellow marker).

Point-to-point response to reviewer comments (underlined by the authors)

Reviewer 1 Advance Summary and Potential Significance to Field: In this manuscript, Yoshida and Hayashi describe in a very interesting epithelial phenotype associated with EGFR depletion in Drosophila embryo. Using a combination of genetics, FRET imaging and live imaging they found that EGFR depletion promotes epithelial collapse in the Drosophila embryo, especially in regions undergoing invagination and detachment from the vitelin membrane. By directly removing the vitelin membrane, they further suggest that EGFR mutant cells are more sensitive to vitelin membrane detachment which provokes massive apoptosis. Epithelial defects not only emerge in regions of invagination, but are also associated with clustered extrusion which depending on the regions can either occur apically (which is atypical in Drosophila, except in the adult midgut) or basally. Overall, many observations are very interesting and open a lot of questions about the relationship between vitelin membrane, cell survival,extrusion, epithelial stability and EGFR. There is however very little mechanistic insights at this stage and most of the putative explanations for the epithelial stability phenotype remain quite hypothetical. I do believe that the fine phenomenology and precise characterization of the phenotype justify a publication by itself, but few genetic experiments may help to clarify/sort some of the explanations proposed by the authors and would make the manuscript more compelling.

We appreciate very much the excitement shown by this reviewer and the kind encouragement to perform additional experiments to deepen the mechanistic insight of this work.

Reviewer 1 Comments for the Author:

1. Three main non-exclusive mechanisms could explain epithelial collapse upon EGFR depletion :

A.High rate of apoptosis and clustered cell death. The high rates of apoptosis may prevent proper execution of extrusion which could lead to sealing defects and catastrophic tissue collapse

B. A deregulation of tension : high tissular tension may promote cell detachment and tissue collapse

C. A deregulation of adhesive forces : a lowering of cell-cell adhesion and/or adhesion to vitelin membrane may weaken cell bound and promotes tissue collapse

Many of these parameters are quite interconnected but some key experiments could be performed to partially sort their contribution:

A.Blocking caspase/apoptosis in the contact of EGFR depletion : could the author overexpress Diap1 or p35, or use the deletion H99 to lower down apoptosis in EGFR mutant background and test if this affect the extrusion events and delay epithelial collapse ? If the genetic is too complicated, the authors may use injection of the pan caspase inhibitor zvad-fmk or even use genetic inhibition of apoptosis combined a drug inhibition of EGFR/ERK provided it phenocopy EGFR null (e.g.: trametinib, a MEK inhibitor, works potently in flies).

B,C.Could the authors provide some comparison of the levels of E-cad and MyoII in the EGFR mutant compared to the control embyos, specially in the region that will collapse first ?

B,C.Is there any evidence of genetic interactions between E-cad, and/or MyoII/Rho1 and EGFR ? (enhancement or partial rescue of the phenotype upon modulation of E-cad, or MyoII activity)

We thank reviewer 1 for summarizing the potential mechanisms of tissue instability in EGFR mutants and suggestions for possible experiments to address them. We attempted to test those ideas.

© 2023. Published by The Company of Biologists under the terms of the Creative Commons Attribution License (https://creativecommons.org/licenses/by/4.0/).

1. Suppressing apoptosis in EGFR mutants to ask if clustered apoptosis triggers tissue disintegration.

We thank this insightful suggestion by reviewer 1. We constructed a strain double mutant for egfr^{f24} and Df(3L)H99 (which deleted proapoptotic genes grim, hid, rpr, ski), each balanced with second and third chromosome balancers with the lacZ markers. Although the stock was very weak, we managed to obtain a few double mutant embryos, which consistently showed very few apoptotic cells and no obvious tissue disintegration. This implies that apoptosis is a prerequisite for the massive tissue disintegration phenotype of EGFR mutants. This new result is presented in revised Fig. 3.

2. To test if cell-cell adhesion and cell contractility are compromised in EGFR mutants, we examined the expression of E-cadherin in EGFR mutants. We noted that the junctional distribution of E-cadherin in epithelial cells of EGFR mutants showed frequent gaps before disintegration, in contrast to the continuous E-cadherin distribution in control epithelial junctions. Subapical junction marker Par6-GFP and the basolateral cell interface marker Dlg remained continuous in EGFR mutants. Those data imply that the integrity of the adherens junction is compromised in EGFR mutants. This new result is presented in Fig. S2.

We also examined the distribution of phosphorylated myosin in EGFR mutants. As previously shown, Myosin distribution is highly dynamic and complex, and we were unable to find a clear change in myosin distribution in EGFR mutants. By no means do we say myosin is unchanged. More in-depth analysis of Myosin dynamics with high-speed live imaging would be required before drawing any conclusion. We want to leave this issue for a future project.

Our new data (revised Fig. 3 and Fig. S2) indicates that the discontinuity of adherens junction may be the basis of reduced surface tension in the EGFR mutant epithelia, causing the rapture of cell junction when excessive cell death in the epithelial invagination site applies pulling tension. This idea is mentioned in the discussion.

2. So far, the authors have shown that vitelin membrane detachment promotes epithelial collapse especially in absence of EGFR. However, it is not clear at this stage whether ERK activation pattern in the WT is related causally to the local invagination and whether vitelin membrane detachment does promote ERK. Actually, movie S1 clearly shows many regions where ERK activation seems to precede tissue invagination and detachment. While it could fit with the notion that EGFR/ERK is required to protect zones of detachment, I would recommend to remain more cautious on the causal link between vitelin membrane detachment and EGFR/ERK activation (as currently stated in the discussion). Unless the authors can show an increase of ERK activation upon vitelin membrane detachement like in Fig 5, I would amend the text whenever this point is mentioned.
Could the author also try to evaluate more systematically whether ERK activation precede / is concomitant with / or comes after the detchament from the vitelin membrane ?

We thank reviewer 1 for raising this point. Invagination, vitelline membrane detachment, and activation of EGFR are, in principle, independently regulated, as we previously reported for the case of tracheal placode invagination where *rhomboid* expression precedes the onset of invagination (Nishimura et al., 2007, Ogura et al., 2018). Consistent with this idea, FRET imaging of a relatively intact, devitellinized stage 11 embryo showed ERK activity in the tracheal pit was maintained at least for 60 minutes, and no ectopic ERK activation was observed in those embryo fragments (new Movie S5). Whether this pattern of ERK followed the normal time course of ERK activity in intact embryos is difficult to evaluate, given the limited imaging capacity for devitellinized embryos. We can say that ERK activity and tissue invagination are separately regulated.

Minor points:

Minor pt 1: Page 2 : sentence : “ promotes de closure of the epithelial opening after apoptotic extrusion (Moreno et al. 2019, Valon et al. 2021)”. I am not sure this is what was shown in these articles (the first one shows that ERK can be modulated by mechanics and

© 2023. Published by The Company of Biologists under the terms of the Creative Commons Attribution License (https://creativecommons.org/licenses/by/4.0/).

the second describes pulses of ERK that change cell death distribution, but non-of them address the role of EGFR for closure and wound healing to my knowledge).

Valon et al. 2021 reported that EGFR depletion in the pupal notum increased the rate of clustered cell elimination (Fig. 5), and this work founds the basis of our work. Moreno et al. 2019 did not directly address the role of EGFR in cell extrusion and will be removed from the citation.

Minor pt 2: The first generation of EKAR can also be sensitive to Cdk1 (which could explain the activation seens during mitosis), see for instance https://www.life-science-alliance.org/content/5/1/e202101206, Lin et al Life science alliance 2021. So that could explain part parttern observed at stage 10 which colocalises with dextran (and has nothing to do with EGFR ERK). Indeed they are still here upon Rhomboid depletion. It is not an essential point for this study but it might be worth mentioning this potential caveat. Along this line, Rhomboid does not really seem to reduce the signal in the groove.

Yes. The EKAR FRET also reports CDK1 activity that should be distinguished from the ERK-invoked FRET activity. CDK1 is active in G1 and mitotic cells. Since G1 is skipped in the embryonic cell cycle 14-16 observed in this study, we must only consider mitotic cells for CDK1-induced EKAR FRET activity. No mitotic figures were observed in the example shown in new Fig. 1C, E, F. Therefore, all the FRET signals in those images can be ascribed to the ERK activity. In the revised Supplemental Figure S1, we show examples of ERK FRET patterns in the control and rhomboid mutants to provide a broad overview of this reporter, including the activity induced in mitotic cells.

Minor pt 3 : In Figure 3F : It was not absolutly clear whether the particle shown with the yellow arrow are indeed Dcp1 negative. It sometime seems like a large cell contains subcompartment that are Dcp1 positive. Do the authors have more example of apical caspase negative cell specially isolated one that can be identified without ambiguity ?

We scanned through the 3D stack images of EGFR mutants stained with Dcp1 and DAPI in this stage and confirmed that the apical tissue masses include Dcp1 negative cells.

Minor pt 4: Could the authors give a sense of the time required to observed tissue collapse upon vitellin membrane removal compared to the time to see collapse in EGFR mutant non- devitellinized embryo ? Is the collapse indeed occuring earlier upon vitelin membrane removal compared to EGFR mutant with vitelin membrane ?

Due to a great difference in culture condition and the extent of morphogenetic movement, it is difficult to compare this time course with what is happening in intact embryos. Therefore we would like to refrain from comparing the temporal order of tissue collapse in the two conditions.

Minor pt 5: It might be good to give more details in the methods on the genotypes. For instance, it was not clear to me whether the egfr f24 mutants were zygotic nulls or maternal/zygotic mutants. It might be worth giving this type of details for all the mutants used in the study.

Throughout this study, the zygotic EGFR phenotype was analyzed because it was previously demonstrated that there is no requirement for the EGFR ligand Spitz in the maternal germline (Mayer and Nüsslein-Volhard, 1988). We stated that zygotic phenotype was studied in this work (page 4, first line of section 2.2).

Reviewer 2 Advance Summary and Potential Significance to Field:

The manuscript from Yoshida and Hayashi describes a study of the involvement of the Drosophila EGF receptor (EGFR) in maintaining epithelial integrity during embryogenesis. EGFR, the most influential receptor mediating signaling via the MAP kinase (MAPK) pathway in Drosophila embryos, participates in a wide range of developmental processes. EGFR embryonic mutant phenotypes are therefore complex, and considerable effort is necessary in order to tease apart the different functional requirements. In this interesting

© 2023. Published by The Company of Biologists under the terms of the Creative Commons Attribution License (https://creativecommons.org/licenses/by/4.0/).

paper, the authors use a range of experimental approaches in order to put forward the novel notion that the EGFR signaling pathway acts to restrict extrusion of (dead) cells to the basal side of epithelia during embryonic morphogenesis. A related notion is that this activity of EGFR is required and stimulated in regions (e.g segmental grooves) where the epidermal epithelium pulls away from the overlying vitelline membrane (VM).

We appreciate the interest and excitement shown by this reviewer.

Reviewer 2 Comments for the Author:
I would like to state at the outset that I encountered considerable difficulty in following the manuscript narrative, which struck me as unfocused and- at times- confusing, and I had to read portions of the text several times, in order to piece together the framework described above. Considerable reorganization is therefore necessary, in my opinion, in order to present the "story" in a substantially more coherent manner. My recommendation is that the authors clarify ahead of time where they are going, describe their work in a fashion that adheres and relates to this stated scenario, and contend better with questions that the data brings up. More specifically:

We regret that the manuscript organization in the early version posed difficulty for reviewer 2 to follow the logic flow. We modified the text as described below.

1. One issue is that the Results section starts off in unconvincing fashion, with an experiment in which a spatial correspondence is claimed between regions (e.g segmental grooves) showing both heightened ERK activity (as monitored with a FRET sensor) and separation between the embryo ectoderm and the vitelline membrane (observed by accumulation of injected Dextran). The authors then attempt to strengthen these rather indirect observations for EGFR function by showing that ERK activity is reduced in rhomboid-1 (rho-1) mutant embryos, which also display a somewhat abnormal measure of apical cell extrusion. This, however, is a weak argument, given the generally mild disruption to morphogenesis observed in rho-1 (as well as in Spitz ligand) mutant embryos, which, in turn, argues for an alternative means of EGFR activation in this context, an issue which the authors themselves raise in the Discussion. I can appreciate the reasoning behind starting the manuscript in this fashion, but it really works against it. My strong recommendation would be to begin with the data and text around Figure 2, stating that the initial motivation for the study is- as already brought up by the authors- a desire to understand the severe morphogenesis phenotype of EGFR mutant embryos, which is not readily explained by currently established roles of this key receptor, and proceed from there.

Thank you very much for pointing out the source of this reviewer's problem in reading the manuscript. We understand that starting with the *rhomboid* phenotype, which is rather weak compared to EGFR, misdirects readers' attention. The revised manuscript starts with the results of the Dextran-FRET data (Original Fig. 1D-G), and proceeds immediately to the description of EGFR mutants. The rhomboid mutant phenotype (original Fig. 1A-C) was removed. Those change does not alter the overall conclusion of this manuscript.

2. The experiments described in Figures 3 and 4 appear to be put forward in order to establish that apical extrusion of cells which have undergone apoptosis, will occur when two conditions are met- epidermis/vitelline membrane (VM) separation and absence of EGFR activity. First off, this needs to be stated clearly.

We have clearly stated this point in the revised manuscript (starting sentence on page 4, section 2.2).
Second- what do the authors think happens in EGFR mutants that initiates and leads to disintegration of the epithelium? They claim to observe that "A gap between the apical cell surface vitelline membrane appeared". Is the idea that this represents a stochastic event with dire consequences in EGFR mutants? Is it a normal event observed in wildtype embryos as well? Does it occur more frequently in the head region (so as to explain the anterior-to- posterior progress of disintegration in the mutants)?

© 2023. Published by The Company of Biologists under the terms of the Creative Commons Attribution License (https://creativecommons.org/licenses/by/4.0/).

Our idea is that the groove formation and clustered mitosis that occur in normal embryogenesis sensitize the epithelia for disintegration when EGFR function is reduced. Detachment of the epithelia from the vitellin membrane is observed at the site of tissue folding and clustered mitosis in normal embryos (Fig. 1, Fig. S1I, I'), and very few cases of apical cell extrusion were observed. We noted any change in the frequency of vitelline membrane detachment in EGFR mutants. Only when the two conditions, detachment of vitelline membrane and the loss of EGFR function, do the epithelia become unstable. We emphasized this point in the discussion.

3. EGFR is well known to function as a “survival factor” that limits apoptosis, and this activity has been shown to be consequential to Drosophila embryonic development (eg- Crossman et al 2018). To what degree does the excessive apoptosis seen in EGFR mutant embryos (and documented in this study as well) contribute to epithelial disintegration? Perhaps the system becomes overwhelmed by excessive cell death? The authors should consider an experiment in which apoptosis is blocked in the EGFR mutant background and assess the consequences and how they reflect on their model.

We thank this insightful suggestion by reviewer 2. As we replied to reviewer 1 comment, we constructed a strain double mutant for $egfr^{f24}$ and Df(3L)H99 (which deleted proapoptotic genes grim, hid, rpr, ski), each balanced with second and third chromosome balancers with the lacZ markers. Although the stock was very weak, we obtained a few double mutant embryos, which consistently showed very few apoptotic cells and no obvious tissue disintegration. This implies that apoptosis is a prerequisite for the massive tissue disintegration phenotype of EGFR mutants. This new result is presented in revised Fig. 3.

4. A couple of remarks regarding data presented in the figures:

-- As discussed above, the data presented in Figure 1 is problematic and not a good way to start the Results section. However, while the rhomboid experiments should, in my opinion, be removed altogether, there is justification for presenting the wildtype FRET sensor and Dextran injection data at a later phase of the text, although not necessarily linked. In any case- if and when presented, the FRET sensor images should be accompanied by a matching “heatmap” schematic or the like, as the pattern of the signal is difficult to make out from the images.

We thank reviewer 1 for this suggestion. We changed the manuscript to start with the results of the Dextran-FRET data (Original Fig. 1D-G), and the rhomboid mutant phenotype (original Fig. 1A-C) was moved to supplement. The heat map of the IMD display used here is indicated at the top of the image.

-- When assessing the consequences of laser wounding (Figure6 E-F), a panel series should be added in which an age-matched unwounded EGFR mutant embryo (expressing Par6-GFP and Histone-RFP) is monitored, to allow comparison of the progress of development in the thorax/abdomen to the wounded embryo.

The embryo shown in Figure 6E, F received a laser shot in the right hemisegment. The contralateral side precisely serves age-matched none-wounded control. Indeed, tissue disintegration starts on the wounded side, and the contralateral segment maintains epithelial integrity, as noted by Par6-GFP imaging. The text was modified to emphasize this point (page 7, first paragraph), as follows “**The initial epidermal disintegration phenotype was limited to the side of laser wounding and took more than 30 minutes to initiate (Fig. 6F)**”.

© 2023. Published by The Company of Biologists under the terms of the Creative Commons Attribution License (https://creativecommons.org/licenses/by/4.0/).

Second decision letter

MS ID#: DEVELOP/2022/201231

MS TITLE: Epidermal growth factor receptor signaling protects epithelia from morphogenetic instability and tissue damage in Drosophila

AUTHORS: Kentaro Yoshida and Shigeo Hayashi
ARTICLE TYPE: Research Article

I am happy to tell you that your manuscript has been accepted for publication in Development, pending our standard ethics checks.

Reviewer 1

Advance summary and potential significance to field

The authors have significantly improved the manuscript by clarifying the link between apoptosis and epithelial collapse, reorganising the manuscript and providing new controls. I fully support publication.

Comments for the author

The authors have significantly improved the manuscript by clarifying the link between apoptosis and epithelial collapse, reorganising the manuscript and providing new controls. I fully support publication.

Reviewer 2

Advance summary and potential significance to field

The manuscript from Yoshida and Hayashi describes a study of the involvement of the Drosophila EGF receptor (EGFR) in maintaining epithelial integrity during embryogenesis. EGFR, the most influential receptor mediating signaling via the MAP kinase (MAPK) pathway in Drosophila embryos, participates in a wide range of developmental processes. EGFR embryonic mutant phenotypes are therefore complex, and considerable effort is necessary in order to tease apart the different functional requirements. In this interesting paper, the authors use a range of experimental approaches in order to put forward the novel notion that the EGFR signaling pathway acts to restrict extrusion of (dead) cells to the basal side of epithelia during embryonic morphogenesis. A related notion is that this activity of EGFR is required and stimulated in regions (e.g segmental grooves) where the epidermal epithelium pulls away from the overlying vitelline membrane (VM).

Comments for the author

In the revised submission, the authors appear to have made genuine efforts to address the comments and concerns I raised in my review of the original manuscript, including reorganization and clarification of the text and addition of a key experiment related to manner in which apoptosis fits into their understanding and data interpretation. My overall assessment of the study remains as before. While interesting notions, based on well-designed and well-performed experiments, are put forward, additional work is clearly required in order to solidify these ideas; still, I believe the study has progressed far enough, so as to be presented to the readership of Development in its current form.

© 2023. Published by The Company of Biologists under the terms of the Creative Commons Attribution License (https://creativecommons.org/licenses/by/4.0/).
